# Inflammasome Activation Dampens Type I IFN Signaling to Strengthen Anti-*Toxoplasma* Immunity

**DOI:** 10.1128/mbio.02361-22

**Published:** 2022-10-10

**Authors:** Zhiqiang Hu, Dan Wu, Jiansen Lu, Yufen Zhang, Shao-Meng Yu, Yingchao Xie, Hongyu Li, Jianwu Yang, De-Hua Lai, Ke Zeng, Huaji Jiang, Zhao-Rong Lun, Xiao Yu

**Affiliations:** a Department of Immunology, School of Basic Medical Sciences, Southern Medical University, Guangzhou, Guangdong, China; b Department of Joint Surgery, Fifth Affiliated Hospital of Southern Medical University, Guangzhou, Guangdong, China; c State Key Laboratory of Biocontrol, School of Life Sciences, Sun Yat-sen University, Guangzhou, Guangdong, China; d Guangdong Provincial Key Lab of Single Cell Technology and Application, Southern Medical University, Guangzhou, Guangdong, China; e Department of Orthopedics, Yue Bei People's Hospital Affiliated to Medical College of Shantou University, Shaoguan, Guangdong, China; University of Calgary

**Keywords:** toxoplasmosis, inflammasome, type I interferon, SOCS1

## Abstract

Innate immunity acts as the first line of defense against pathogen invasion. During Toxoplasma gondii infection, multiple innate immune sensors are activated by invading microbes or pathogen-associated molecular patterns (PAMPs). However, how inflammasome is activated and its regulatory mechanisms during T. gondii infection remain elusive. Here, we showed that the infection of PRU, a lethal type II T. gondii strain, activates inflammasome at the early stage of infection. PRU tachyzoites, RNA and soluble tachyzoite antigen (STAg) mainly triggered the NLRP3 inflammasome, while PRU genomic DNA (gDNA) specially activated the AIM2 inflammasome. Furthermore, mice deficient in AIM2, NLRP3, or caspase-1/11 were more susceptible to T. gondii PRU infection, and the ablation of inflammasome signaling impaired antitoxoplasmosis immune responses by enhancing type I interferon (IFN-I) production. Blockage of IFN-I receptor fulfilled inflammasome-deficient mice competent immune responses as WT mice. Moreover, we have identified that the suppressor of cytokine signaling 1 (SOCS1) is a key negative regulator induced by inflammasome-activated IL-1β signaling and inhibits IFN-I production by targeting interferon regulatory factor 3 (IRF3). In general, our study defines a novel protective role of inflammasome activation during toxoplasmosis and identifies a critical regulatory mechanism of the cross talk between inflammasome and IFN-I signaling for understanding infectious diseases.

## INTRODUCTION

Toxoplasmosis, caused by Toxoplasma gondii, is one of the most prevalent parasitic diseases worldwide, affecting large numbers of warm-blooded animals, covering approximately a third of the world's human population (1). Toxoplasmosis can lead to serious illness in humans of all ages, especially in immunosuppressed patients, neonates, and pregnant women. T. gondii infection instigates a range of symptoms, manifesting as acute and chronic infection, including lymphadenitis, ocular disease, central nervous system (CNS) damage, and abortion (2–4). Although big progress has been achieved in many fields of T. gondii in the last decades, many host immune responses against this parasite infection are still urgent to be well understood.

It is well known that the innate immune system plays an essential role in recognizing PAMPs secreted by T. gondii, including profilin, cyclophilin, dense granule (GRA), rhoptry (ROP), and GPI-anchored (GPI-A) proteins, and leads to multiple immune responses to control infection (5–7). Upon T. gondii infection, host pattern recognition receptors (PRRs) trigger the activation of nuclear factor κB (NF-κB), type I interferon (IFN-I), Janus kinase (JAK)-signal transducer and activator of transcription (STAT), and inflammasome pathways (8–12). Accumulating evidence supports that these signaling pathways need to be accurately and restrictedly controlled, which would contribute tremendously to limiting *Toxoplasma* replication (13–16), but little is known about the regulation and cross talk between these signaling pathways during T. gondii infection.

Inflammasomes are cytosolic multimeric protein complexes activated by microbial molecules or stress signals which display multiple-step processing for caspase-1 dimerization and production of mature IL-1β and IL-18 (17). During T. gondii infection, inflammasomes, including NLRP1, NLRP3, and AIM2, contribute to host defense together, but they are distinct in terms of ligand binding, complex composition, and activation mechanisms (18). In Lewis rat bone marrow-derived macrophages (BMDMs), activation of T. gondii-mediated NLRP1 inflammasome mainly depends on the release of dense granule proteins, including GRA35, GRA42, and GRA43 (19). However, mechanisms of NLRP1b activation in murine and human cells during T. gondii infection are unclear. In contrast to the dispensable role of NLRP3 for T. gondii infection in rats, both NLRP1 and NLRP3 are important for controlling T. gondii in mice (20). Many results indicated that inducements, like potassium K^+^ efflux, ATP, reactive oxygen species (ROS), and cleavage of caspase-1/11, could lead to NLRP3 activation during T. gondii infection (21–23). Despite the vital role of AIM2 in viral infection (24–26), studies regarding the effect of AIM2 on T. gondii infection were limited, and even AIM2 has been reported to induce gasdermin D (GSDMD)-independent, apoptosis-associated speck-like protein containing a caspase-recruitment domain (ASC)- and caspase-8-dependent apoptosis through detecting T. gondii DNA during toxoplasmosis (27). Although many studies are focused on this field, inflammasome activation and its effect on host immunity during the lethal type II T. gondii PRU infection remain elusive.

Compared to inflammasomes, studies about the function of IFN-I in T. gondii infection are limited. An early study suggested that IFN-I production during T. gondii infection required three fundamental events: parasite internalization, toll-like receptor (TLR) activation, and efficient MyD88 signaling (28), but no definitive experiments have proven these processes. A recent report indicated that T. gondii infection triggered cGAS/STING signaling and led to IFN-I production (29). Although the evidence that IFN-I signaling is activated during *Toxoplasma* infection is confirmed, the role of IFN-I is controversial in different infection models. IFN-I has been reported to suppress T. gondii growth in the oral cyst infection model; however, the production of IFN-I in the host was moderate (30, 31). On the contrary, IRF3, the key element of TBK1-IFN-I signaling, was reported to promote replication of T. gondii (32, 33). In addition, IFN-I secretion could be suppressed through distinct mechanisms (32, 34, 35). Even then, how IFN-I is activated is unknown. Therefore, it is urgent to understand the mechanisms underlying IFN-I regulation and its role during T. gondii infection.

In this study, we sought to investigate the mechanisms beyond inflammasome activation and its effect on host anti-T. gondii immunity. In the lethal PRU strain (36–39), the infection can trigger inflammasome activation both *in vivo* and *in vitro*. Moreover, *in vitro* PAMPs stimulation showed NLRP3 inflammasomes were activated by PRU RNA and STAg, while AIM2 activation mainly depends on PRU gDNA. Activation of inflammasome promoted anti-T. gondii immunity by suppressing IFN-I production, which played a detrimental role in generating immunity against T. gondii infection. We further demonstrated that inflammasome activation induced SOCS1 expression, thus inhibiting the IFN-I signaling pathway by targeting IRF3. Our findings suggest an unrecognized regulatory mechanism of inflammasome signaling in IFN-I response in anti-*Toxoplasma* immunity and highlight the cross talk between inflammasome and IFN-I signaling in modulating immune responses against T. gondii infection.

## RESULTS

### T. gondii infection activates the inflammasome and leads to IL-1β production.

**FIG 1:**
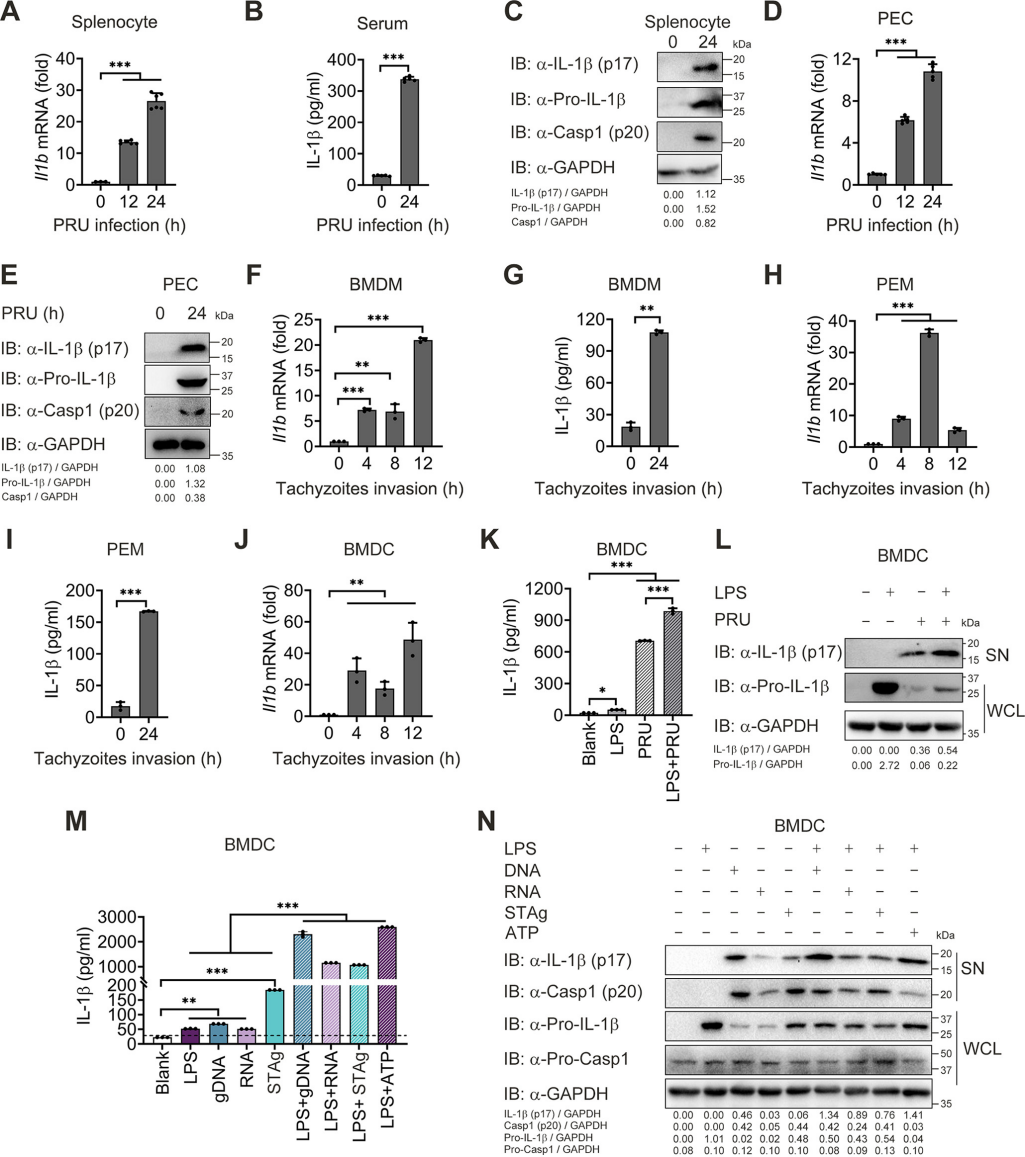
Toxoplasma gondii infection and its associated PAMPs stimulation activate inflammasome and result in IL-1β secretion. (A to E) WT mice (*n* = 5) were intraperitoneally infected with T. gondii (1 × 10^5^ PRU tachyzoites). Splenocytes and PECs were collected at indicated times postinfection, then subjected to a qRT-PCR test for *Il1b* mRNA levels (A and D), or immunoblotting analysis (C and E), and IL-1β levels in the sera of mice were determined by ELISA (B). (F to I) Primary BMDM and PEM obtained from WT mice were infected with tachyzoites at MOI = 3 for 0, 4, 8, 12, and 24 h. *Il1b* and *Gapdh* mRNA were quantified by qRT-PCR (F and H), and IL-1β levels in the cell supernatants were determined by ELISA (G and I). (J) BMDC isolated from WT mice were infected with tachyzoites at MOI = 3 for 0, 4, 8, and 12 h, then subjected to a qRT-PCR test for *II1b* mRNA levels. (K and L) BMDC were infected with tachyzoites for 18 h at MOI = 3 with or without LPS (500 ng/mL) pretreatment for 2 h, and total IL-1β secreted in supernatants were determined by ELISA (K), and mature IL-1β (p17) in supernatants or Pro-IL-1β in whole-cell lysates were determined by immunoblotting analysis (L). (M and N) WT BMDCs were stimulated by T. gondii-associated PAMPs, including gDNA, RNA, or STAg, for 18 h with or without LPS (500 ng/mL) pretreatment for 2 h, and supernatants were collected for quantization of IL-1β cytokine by ELISA analysis (M), and cell lysates along with supernatants were collected for immunoblotting analysis with indicated antibodies (N). Data are representative of three independent experiments and plotted as mean ± SD. *, *P < *0.05; **, *P < *0.01; ***, *P < *0.001 versus corresponding control. See also Fig. S1.

Previous studies showed that inflammasome activation and IL-1β production played a vital role in anti-*Toxoplasma* immunity (18, 22, 40, 41). However, it is not clear when inflammasome is activated post-*Toxoplasma* infection. To assess the inflammasome response during the lethal T. gondii infection *in vivo*, wild-type (WT) C57BL/6 mice were administered the tachyzoites of the lethal T. gondii PRU strain intraperitoneally, and splenocytes were collected for detection of inflammasome activation. We found a significant increase of *Il1b* mRNA at 12 and 24 h postinfection (hpi) (Fig. 1A). Besides, protein levels of IL-1β in the serum, along with mature IL-1β (p17) and the cleaved fragment of procaspase-1 (p10), boosted obviously at 24 hpi (Fig. 1B and C). Similar results were obtained in the peritoneal cells (PECs) from T. gondii-infected mice (Fig. 1D and E), suggesting that inflammasome signaling was activated at the early stage of toxoplasmosis.

10.1128/mbio.02361-22.1 FIG S1 T. gondii-associated PAMPs stimulation activates IL-1β production and secretion in primary mouse cells. (Related to Fig. 1) (A to C) BMDM (A), PEM (B), and BMDC (C) were stimulated with STAg for indicated time points, and mRNA levels for *Il1b* were measured by using qRT-PCR. (D to F) BMDM (D), PEM (E), and BMDC (F) were stimulated with gDNA for indicated time points, and mRNA levels for *Il1b* were measured by using qRT-PCR. (G to I) BMDM (G), PEM (H), and BMDC (I) were stimulated with RNA for indicated time points, and mRNA levels for *Il1b* were measured by using qRT-PCR. (J and K) WT PEM was stimulated by T. gondii associated PAMPs for 18 h with or without LPS (500 ng/mL) pretreatment for 2 h, then supernatants were collected for determination of IL-1β cytokine by ELISA analysis (J), and cell lysates and supernatants were collected for immunoblotting analysis (K). Data are representative of three independent experiments and plotted as mean ± SD. *, *P < *0.05; **, *P < *0.01; ***, *P < *0.001 versus corresponding control. Download FIG S1, TIF file, 0.7 MB. Copyright © 2022 Hu et al. 2022 Hu et al. https://creativecommons.org/licenses/by/4.0/ This content is distributed under the terms of the Creative Commons Attribution 4.0 International license.

To further verify inflammasome activation *in vitro*, isolated primary mouse BMDMs, PEMs, and bone marrow dendritic cells (BMDCs) were infected with tachyzoites of PRU for indicated times. Consistent with the phenomenon *in vivo*, the transcriptional level of *Il1b* and protein released in the supernatant was detected to a rising tendency post-PRU tachyzoites invasion in BMDMs, peritoneal macrophages (PEMs) and BMDCs (Fig. 1F to J). Further studies exhibited that PRU tachyzoites infection caused an enhanced IL-1β response when NF-κB signaling was preactivated by LPS in BMDCs (Fig. 1K and L). These data suggest that T. gondii invasion could activate inflammasome signaling *in vitro*.

Nonetheless, it remains undetermined how the inflammasome is activated. To figure out this, we purified different PAMPs from T. gondii and stimulated BMDMs, PEMs, and BMDCs with associated PAMPs. We found that STAg (see Fig. S1A to C in the supplemental material), gDNA (Fig. S1D to F), and RNA (Fig. S1G to I) from PRU triggered continuous *Il1b* mRNA expression. Meanwhile, gDNA, RNA, and STAg alone could induce a low production of IL-1β, which could be strengthened visibly in LPS-primed BMDCs and PEMs (Fig. 1M and N, Fig. S1J and K). Altogether, these results suggest that T. gondii tachyzoites and associated PAMPs could activate inflammasomes and lead to IL-1β production at the early stage of T. gondii infection.

### T. gondii tachyzoites, RNA, and STAg activate the NLRP3 inflammasome, while gDNA triggers AIM2 inflammasome activation.

**FIG 2:**
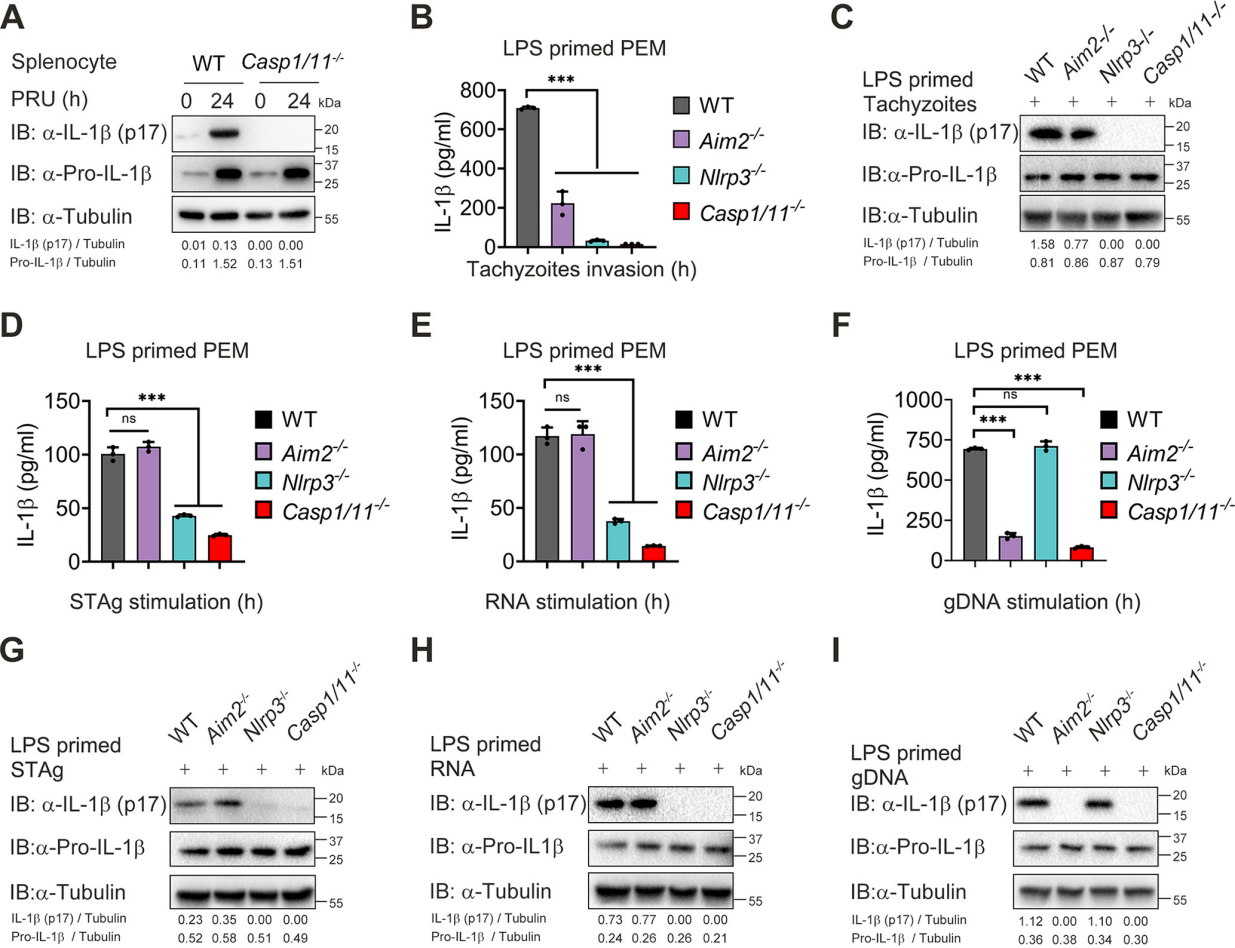
T. gondii tachyzoites, RNA, and STAg, activate the NLRP3 inflammasome, while gDNA triggers AIM2 inflammasome activation. (A) WT and *Casp1/11^−/−^* mice (*n* = 4) were intraperitoneally injected with T. gondii (1 × 10^5^ PRU tachyzoites), and splenocytes were harvested at 24 h after infection. Uninfected samples served as the control group. Cell lysates were analyzed by immunoblotting with the indicated antibodies and shown with a respective image. (B and C) LPS-primed WT, *Aim2^−/−^*, *Nlrp3^−/−^*, and *Casp1/11^−/−^* PEMs were infected with PRU tachyzoites at MOI = 3 for 18 h, and supernatants were collected for quantization of IL-1β cytokine by ELISA analysis (B), and proteins within cell lysates along with supernatants were collected for immunoblotting analysis (C). (D to I) LPS-primed WT, *Aim2^−/−^*, *Nlrp3^−/−^*, and *Casp1/11^−/−^* PEM were stimulated with STAg (D and G), RNA (E and H), or gDNA (F and I) for 18 h, and supernatants were collected for quantization of IL-1β cytokine by ELISA analysis (D and F), and proteins within cell lysates and supernatants were collected for immunoblotting analysis (G and I). Data are representative of three independent experiments and plotted as mean ± SD. *, *P < *0.05; **, *P < *0.01; ***, *P < *0.001 versus corresponding control.

Previous studies showed that T. gondii dense granule proteins GRA35, GRA42, and GRA43 contributed to NLRP1 inflammasome activation, and NLRP3 could be activated through multiple mechanisms during T. gondii infection (19, 42, 43). However, the role of inflammasomes, especially AIM2, and their downstream signaling molecules in the lethal T. gondii infection remains to be defined. To this end, we first compared inflammasome activation in WT and *Casp1/11^−/−^* mice after PRU infection and found that cleavage of IL-1β was completely abolished in *Casp1/11^−/−^* splenocytes at 24 hpi (Fig. 2A), suggesting that downstream molecule caspase-1/11 is essential for inflammasome activation induced by T. gondii.

Next, we infected PEMs extracted from mice deficient in AIM2, NLRP3, or caspase-1/11 with PRU tachyzoites and found that IL-1β production and cleavage of pro-IL-1β in the PEMs from *Aim2^−/−^* mice were markedly lower than those from WT mice, while we almost could not detect mature IL-1β and cleavage of pro-IL-1β in the PEMs from *Nlrp3^−/−^* and *Casp1/11^−/−^* mice (Fig. 2B and C). To better understand the contribution of NLRP3 and AIM2, we further stimulated PEMs with RNA, STAg, or gDNA of PRU. As expected, deficiency in NLRP3 or caspase-1/11 significantly affected RNA- or STAg-triggered IL-1β maturation, whereas lacking AIM2 or caspase-1/11 reduced gDNA-triggered IL-1β maturation (Fig. 2D to I). Together, these results indicate that the inflammasome response triggered by gDNA depends on AIM2, and NLRP3 is essential to RNA and STAg recognition during T. gondii infection.

### Inflammasome activation plays a protective role in host defense against T. gondii.

**FIG 3:**
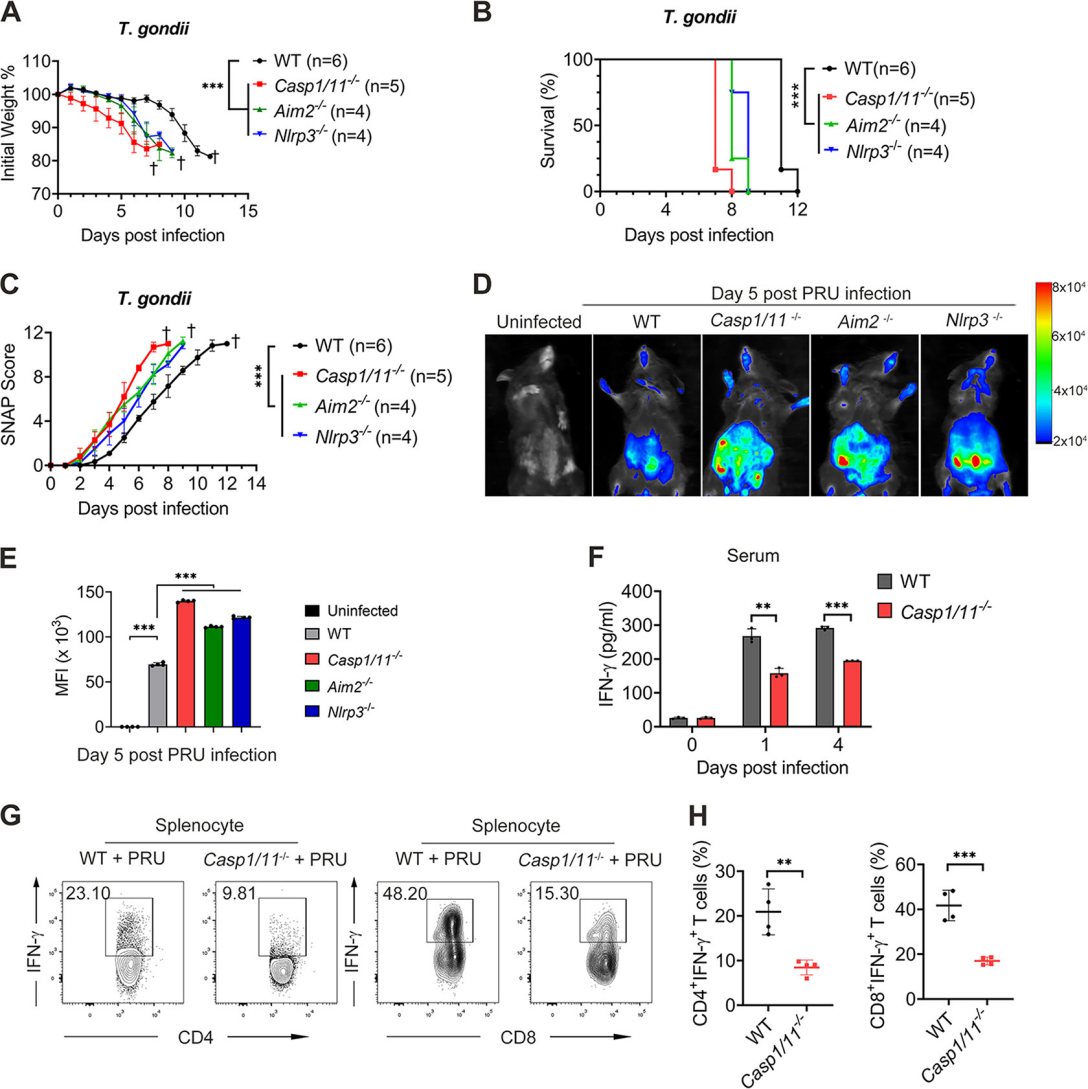
Inflammasome activation plays a protective role in host defense against T. gondii immune response. (A to C) WT, *Casp1/11^−/−^*, *Aim2^−/−^*, and *Nlrp3^−/−^* mice (indicated numbers for each group) were intraperitoneally infected with T. gondii (1 × 10^4^ PRU tachyzoites). Daily changes in body weight (A), survival rates (B), and SNAP scores (C) were recorded. (D and E) WT, *Casp1/11^−/−^*, *Aim2^−/−^*, and *Nlrp3^−/−^* mice (*n* = 3 for each group) were intraperitoneally infected with T. gondii expressing RFP (1 × 10^4^ PRU tachyzoites), and parasite burden was measured at 5 days postinfection by bioluminescence imaging (D), and mean fluorescence intensity (MFI) were as shown (E). (F to H) WT and *Casp1/11^−/−^* mice (*n* = 3 for each group) were intraperitoneally infected with T. gondii (1 × 10^5^ PRU tachyzoites), and the serum was subjected to ELISA of IFN-γ at indicated time points (F), and mice were sacrificed at day 5 postinfection, then splenocytes were collected for the test of percentages of IFN-γ^+^CD4^+^ T cells and IFN-γ^+^CD8^+^ T cells using flow cytometry (G and H). Data are representative of three independent experiments and plotted as mean ± SD. *, *P < *0.05; **, *P < *0.01; ***, *P < *0.001 versus corresponding control. Dagger denotes mouse death. See also Fig. S2.

Next, we continued to determine the *in vivo* role of the inflammasome during PRU acute infection. To address this issue, we infected WT, *Aim2^−/−^*, *Nlrp3^−/−^*, and *Casp1/11^−/−^* mice with a lethal dosage of PRU tachyzoites and found that the mice deficient in AIM2, NLRP3, or caspase-1/11 were more susceptive upon lethal PRU acute infection. *Aim2^−/−^*, *Nlrp3^−/−^*, and *Casp1/11^−/−^* mice had lower body weight and shorter survival time than WT mice (Fig. 3A and B). Furthermore, mice deficient in these inflammasomes held higher simple neuroassessment of asymmetric impairment (SNAP) scores, a functional defect assessment, including several behavioral tests (44) (Fig. 3C). Consistently, we also found the parasite proliferated markedly faster in *Aim2^−/−^*, *Nlrp3^−/−^*, and *Casp1/11^−/−^* mice (Fig. 3D and E).

10.1128/mbio.02361-22.2 FIG S2 Activation of inflammasome plays a protective role in host defense against T. gondii immune response. (Related to Fig. 3) FACS gating strategy for CD4^+^IFN-γ^+^ T cells and CD8^+^IFN-γ^+^ T cells from splenocytes. Download FIG S2, TIF file, 0.8 MB. Copyright © 2022 Hu et al. 2022 Hu et al. https://creativecommons.org/licenses/by/4.0/ This content is distributed under the terms of the Creative Commons Attribution 4.0 International license.

Since IFN-γ has been verified vital for defending against T. gondii infection (45, 46), we next detected the expression of IFN-γ in WT and *Casp1/11^−/−^* mice after lethal PRU challenge. Compared with WT mice, serum level of IFN-γ decreased dramatically in *Casp1/11^−/−^* mice on days 1 and 4 after PRU infection (Fig. 3F). In addition, the percentages of splenic IFN-γ^+^CD4^+^ and IFN-γ^+^CD8^+^ cells from *Casp1/11^−/−^* mice were markedly lower than those from WT mice (Fig. 3G and H, Fig. S2). Altogether, these data indicate that inflammasome activation is required for protecting the host against T. gondii infection.

### Inflammasome activation dampens production of type I IFN cytokines.

We next sought to determine how inflammasome-deficient mice generate defective immune responses against PRU infection. Our previous studies showed that inflammasome activation negatively regulates type I IFN signaling, which weakens antimalaria innate immunity (47). Hence, we sought to explore whether this mechanism during *Plasmodium* infection exists in anti-T. gondii immunity as well. We first traced IFN-β expression and IFN-I signaling activation in WT mice infected with PRU tachyzoites for the indicated times and found the production of *Ifnb* mRNA and phosphorylation of TBK1 were enhanced at 24 h post-PRU infection (Fig. S3A to C). PRU tachyzoites, gDNA, and RNA stimulation were also found to trigger IFN-β production in BMDMs and PEMs (Fig. S3D to I), suggesting that PRU infection could activate type I IFN at an early stage of invasion or stimulation.

**FIG 4:**
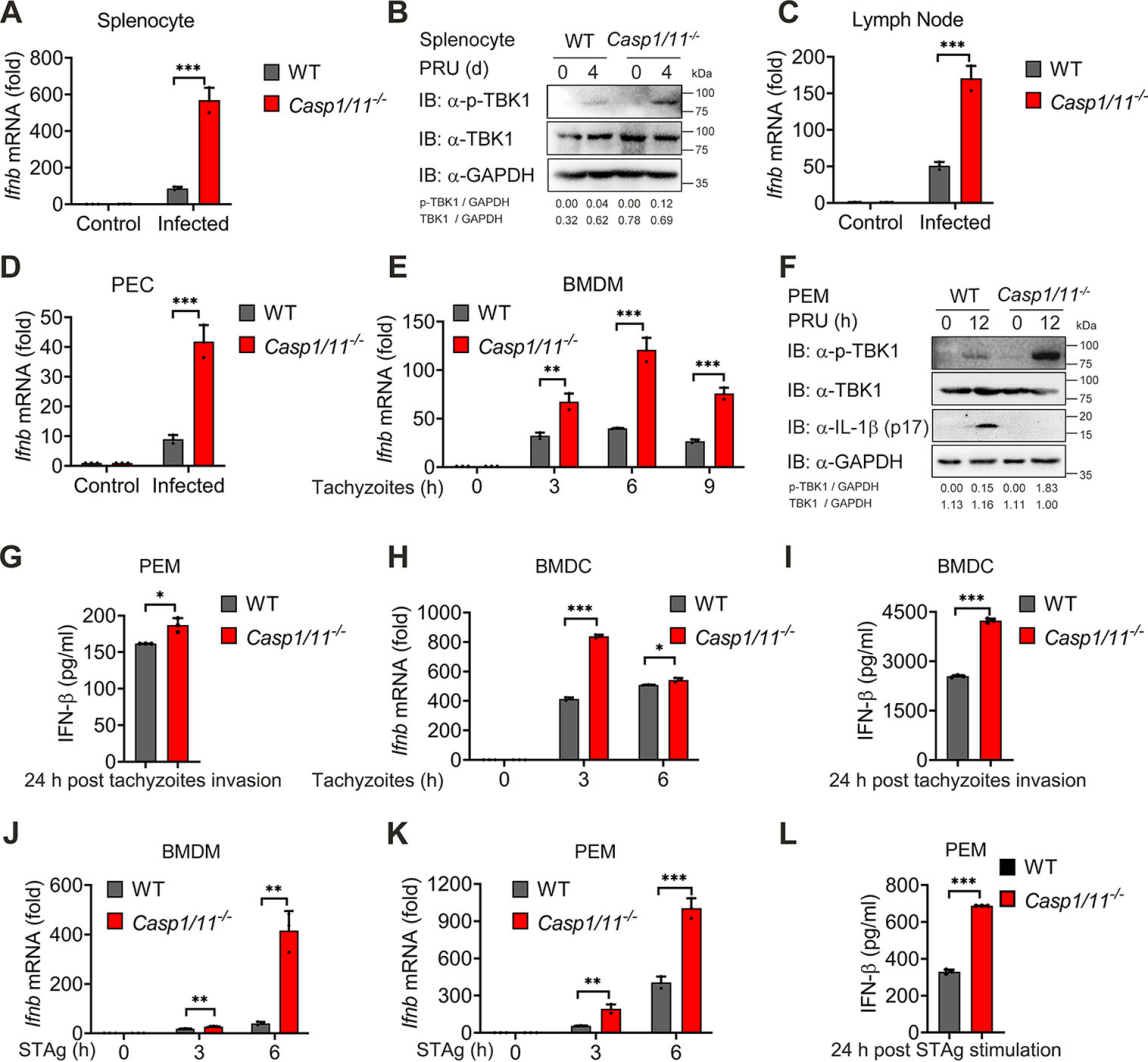
Inflammasome activation dampens type I IFN cytokine production. (A to D) WT and *Casp1/11^−/−^* mice (*n* = 3) were intraperitoneally infected with T. gondii (1 × 10^5^ PRU tachyzoites), and splenocytes harvested at indicated times were used for quantifying the expression of *Ifnb* by using qRT-PCR (A) and immunoblotting analysis (B) with indicated antibodies. Lymph nodes (C) or PECs (D) were collected and subjected to quantifying the expression of *Ifnb* by qRT-PCR. (E) WT and *Casp1/11^−/−^* BMDMs were infected with PRU tachyzoites at MOI = 3 for 0, 3, 6, and 9 h *in vitro*; RNA isolated from BMDMs were used for expression analysis of *Ifnb* by using qRT-PCR. (F and G) WT and *Casp1/11^−/−^* PEMs infected with PRU tachyzoites at MOI = 3 for 12 or 24 h were used for expression analysis of *Ifnb* by using qRT-PCR (F), and IFN-β levels were detected by ELISA in the supernatants (G). (H and I) WT and *Casp1/11^−/−^* BMDC infected with PRU tachyzoites at MOI = 3 for 0, 3, and 6 h were used for expression analysis of *Ifnb* by using qRT-PCR (H) and IFN-β secreted in supernatants were detected by ELISA (I). (J and L) WT and *Casp1/11^−/−^* BMDM (J) or PEM (K) were stimulated with STAg for indicated time points, then subjected to quantifying the expression of *Ifnb* by qRT-PCR. IFN-β levels in the PEM supernatants were determined by ELISA (L). Data are representative of three independent experiments and plotted as mean ± SD. *, *P < *0.05; **, *P < *0.01; ***, *P < *0.001 versus corresponding control. See also Fig. S3.

10.1128/mbio.02361-22.3 FIG S3 Type I IFN signaling can be activated by T. gondii infection and associated PAMPs stimulation in vivo and in vitro. (Related to Fig. 4) (A to C) WT mice were intraperitoneally infected with T. gondii (1 × 10^5^ PRU tachyzoites). (A) mRNA levels for *Ifnb* in the splenocytes from infected WT mice at indicated time points using qRT-PCR. (B) IFN-β levels in the sera from mice infected or not at indicated time points were measured by ELISA. (C) pTBK1 and TBK1 protein expression in the splenocytes of mice infected or not at indicated time points were detected using western blot analysis. (D and E) BMDMs isolated from WT mice were infected with PRU tachyzoites at MOI = 3 for indicated times, RNA was isolated and used for the expression analysis of *Ifnb* by using qRT-PCR (D), and supernatants were collected at 24 h postinfection for tracking IFN-β secretion using ELISA (E). (F to I) WT BMDM were stimulated with gDNA (F and G) or RNA (H and I) for indicated times, then RNA was isolated and used for the expression analysis of *Ifnb* by using qRT-PCR (F and H), and supernatants were collected at 24 h postinfection for tracking IFN-β secretion using ELISA (G and I). (J) WT and *Casp1/11^−/−^* BMDM were stimulated with gDNA and RNA for 24 h, and supernatants were collected for tracking IFN-β secretion using ELISA. (K) THP-1 cells were infected with PRU tachyzoites for 24 h with or without caspase-1 specific inhibitor VX765 treatment, and supernatants were collected for tracking IFN-β secretion using ELISA. Data are representative of three independent experiments and plotted as mean ± SD. *, *P < *0.05; **, *P < *0.01; ***, *P < *0.001 versus corresponding control. Download FIG S3, TIF file, 0.5 MB. Copyright © 2022 Hu et al. 2022 Hu et al. https://creativecommons.org/licenses/by/4.0/ This content is distributed under the terms of the Creative Commons Attribution 4.0 International license.

To investigate whether inflammasome activation affects type I IFN response during PRU infection, we compared IFN-I production between WT and *Casp1/11^−/−^* mice infected with PRU and found that the IFN-β expression in splenocytes was enhanced when caspase-1/11 was deficient, along with a marked increase in TBK1 phosphorylation (Fig. 4A and B). Consistent data were also obtained in the lymph nodes (Fig. 4C). To further identify this phenomenon, we infected BMDMs with PRU tachyzoites *in vitro* and noted that levels of IFN-β mRNA and protein, as well as IFN-I signaling activation, were enhanced significantly when inflammasome signaling was abolished (Fig. 4D to F, Fig. S3J). Similar experiments performed in PEMs, BMDMs, and BMDCs with tachyzoites invasion or STAg stimulation also came to the same conclusion (Fig. 4G to L). Furthermore, we also used caspase-1-specific inhibitor VX765 to inhibit inflammasome signaling in THP-1 cells and found that the production of IFN-β in the supernatants enhanced when inflammasome signaling was blocked by VX765 at 24 h after tachyzoites invasion (Fig. S3K). Altogether, these data suggest that the activation of inflammasome signaling inhibits IFN-I production at an early stage of T. gondii infection.

### Type I IFN is detrimental to anti-T. gondii immune response.

**FIG 5:**
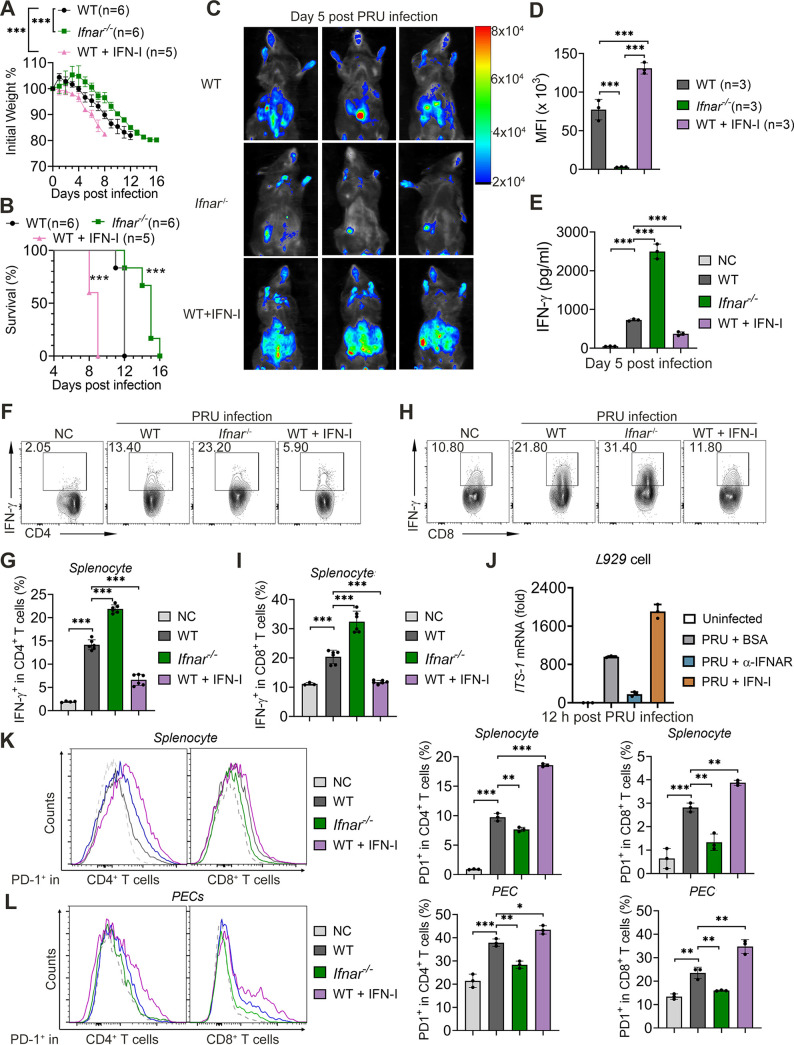
Type I IFN activated by T. gondii infection or associated PAMPs is detrimental to anti-T. gondii immune responses. (A to C) WT (*n* = 6), *Ifnar^−/−^* (*n* = 6), and IFN-I treated WT mice (*n* = 5) were intraperitoneally infected with T. gondii (1 × 10^4^ PRU tachyzoites). Daily changes in body weight (A) and survival rates (B) were recorded. (C and D) WT, *Ifnar^−/−^*, and IFN-I-treated WT mice (*n* = 3 for each group) were intraperitoneally infected with T. gondii expressing RFP (1 × 10^4^ PRU tachyzoites), and parasite burden was measured at 5 days postinfection by bioluminescence imaging (C) and MFI (D) were as shown. (E to I) WT and *Ifnar^−/−^* mice were intraperitoneally infected with T. gondii (1 × 10^5^ PRU tachyzoites), then serum was subjected for measurement of levels of IFN-γ using ELISA (E), and splenocytes collected on day 5 postinfection were subjected to test for the percentages of IFN-γ^+^CD4^+^ T cells (F and G) and IFN-γ^+^CD8^+^ T cells (H and I) using flow cytometry. (J) L929 cells were treated with BSA, anti-IFNAR, or IFN-I, respectively, which were simultaneously infected using PRU expressing RFP for 12 h. The parasite load within cells was determined by qRT-PCR. (K and L) WT, *Ifnar^−/−^*, and IFN-I-treated WT mice were intraperitoneally infected with T. gondii (1 × 10^5^ PRU tachyzoites), and splenocytes along with PECs collected on day 5 postinfection were subjected to test for the percentages of PD1^+^CD4^+^ T cells (K) and PD1^+^CD8^+^ T cells (L) using flow cytometry. Data are representative of three independent experiments and plotted as mean ± SD. *, *P < *0.05; **, *P < *0.01; ***, *P < *0.001 versus corresponding control. Dagger denotes mouse death. See also Fig. S4.

IFN-I has been reported to be vital for innate immunity, especially for defending against the virus, but the role of IFN-I during toxoplasmosis is elusive and controversial (28, 31–33, 35). To identify how IFN-I functions on antitoxoplasmosis immunity, particularly for intraperitoneal infection using PRU strain, we first infected WT and *Ifnar*^−/−^ mice with PRU tachyzoites and found that the absence of the interferon-α/β receptor (IFNAR) could rescue host survival and weight loss during T. gondii infection, which was also presented with fewer parasites load, slighter splenomegaly, and lower SNAP scores compared to WT mice (Fig. 5A to D, Fig. S4A to C). In contrast, WT mice treated with IFN-I on days 0 and 2 postinfection failed to generate strong immunity against PRU infection, resulting in severe weight loss, more parasite loads, serious splenomegaly, high SNAP scores, and lower mortalities within 9 days postinfection (Fig. 5A to D, Fig. S4A to C). Additionally, we tracked a larger amount of IFN-γ in the serum of *Ifnar*^−/−^ mice than those found in WT mice, while mice treated with IFN-I generated fewest IFN-γ during PRU infection (Fig. 5E). Consistently, FACS assay showed that IFN-γ^+^CD4^+^ T cells and IFN-γ^+^CD8^+^ T cell populations increased in the splenocytes of IFNAR deficient mice compared with those populations in WT mice, but a supplement of exogenous IFN-I suppressed this function of T cells (Fig. 5F to I). Furthermore, the recombinant IFN-I promoted *T. gondii* growth in L929 cells, but blocking IFNAR slowed down the proliferation of tachyzoites compared to the BSA group (Fig. 5J, Fig. S4D). These findings suggest that IFN-I induced by T. gondii infection deteriorates host antitoxoplasmosis immunity via dampening T-cell function and promoting T. gondii growth.

10.1128/mbio.02361-22.4 FIG S4 Type I IFN activated by T. gondii infection or its associated PAMPs is harmful to anti-T. gondii immune response. (Related to Fig. 5) (A and B) WT, *Ifnar^−/−^* and IFN-I treated WT mice (each group with indicated numbers) were intraperitoneally infected with T. gondii (1 × 10^5^ PRU tachyzoites), and sacrificed at day 5 postinfection, the respective spleen images were presented (A) and subjected for spleen index analysis (B). (C) WT (*n* = 6), *Ifnar^−/−^* (*n* = 6) and IFN-I treated WT mice (*n* = 5) were intraperitoneally infected with T. gondii (1 × 10^4^ PRU tachyzoites), daily SNAP scores were recorded. (D) L929 cells were treated with BSA, anti-IFNAR, or IFN-I, respectively, infected using PRU expressing RFP for 12h at MOI = 3, and the parasite load within cells was determined by fluorescence image. (E to G) WT, *Ifnar^−/−^* and IFN-I treated WT mice were intraperitoneally infected with T. gondii (1 × 10^5^ PRU tachyzoites), and splenocytes along with PECs collected on day 5 postinfection were subjected to test for the percentages of CD4^+^Foxp3^+^ cells (E) and CD11b^+^Ly6G^+^/CD11b^+^Ly6C^+^ cells (F) using flow cytometry, and the splenocytes along with PECs were also subjected to qRT-PCR for *Pdcd1*, *Siglec15*, *Tim3*, *Lag3*, and *Ctla4* expression analysis **(G)**. Data are representative of three independent experiments and plotted as mean ± SD. *, *P < *0.05; **, *P < *0.01; ***, *P < *0.001 versus corresponding control. The scale bar indicates 100 μm, and dagger denotes mouse death. Download FIG S4, TIF file, 3.7 MB. Copyright © 2022 Hu et al. 2022 Hu et al. https://creativecommons.org/licenses/by/4.0/ This content is distributed under the terms of the Creative Commons Attribution 4.0 International license.

To understand how IFN-I inhibits T-cell immunity, we then expanded our research to classical suppressive immune cells, such as T-regulatory cells (Tregs) and myeloid-derived suppressor cells (MDSCs) (48–51). Nevertheless, we found no differences in the Treg cells and MDSCs of WT, *Ifnar^−/−^*, and IFN-I treated WT mice, though these two cell populations raised with PRU infection, suggesting that Treg cells and MDSCs are not responsible for inhibiting the protective immunity against PRU infections that IFN-I caused (Fig. S4E and F). Moreover, we further examined the expression of T-cell suppression and exhaustion surface markers from splenocytes along with PECs and found only the mRNA levels of *Pdcd1* (PD-1), but not *Tim3*, *Lag3*, and *Ctla4*, increased, and the transcriptional levels of *Pdcd1* were significantly upregulated in IFN-I treated WT mice. However, *Ifnar^−/−^* mice showed a limited PD-1 expression compared to the other two groups (Fig. S4G). Similarly, PD-1^+^CD4^+^ and PD-1^+^CD8^+^ T cells were also increased in the splenocytes, and peritoneal cells (PECs) isolated from IFN-I treated mice with PRU infection. However, these cell populations were markedly decreased within PRU-infected IFNAR knockout mice compared with those in WT mice PRU infected (Fig. 5K and L). Generally, these results suggest that the IFN-I destroys the protective immunity against PRU infections by facilitating the expression of PD-1.

### Inflammasome enhances anti-T. gondii immunity via suppressing IFN-I.

**FIG 6:**
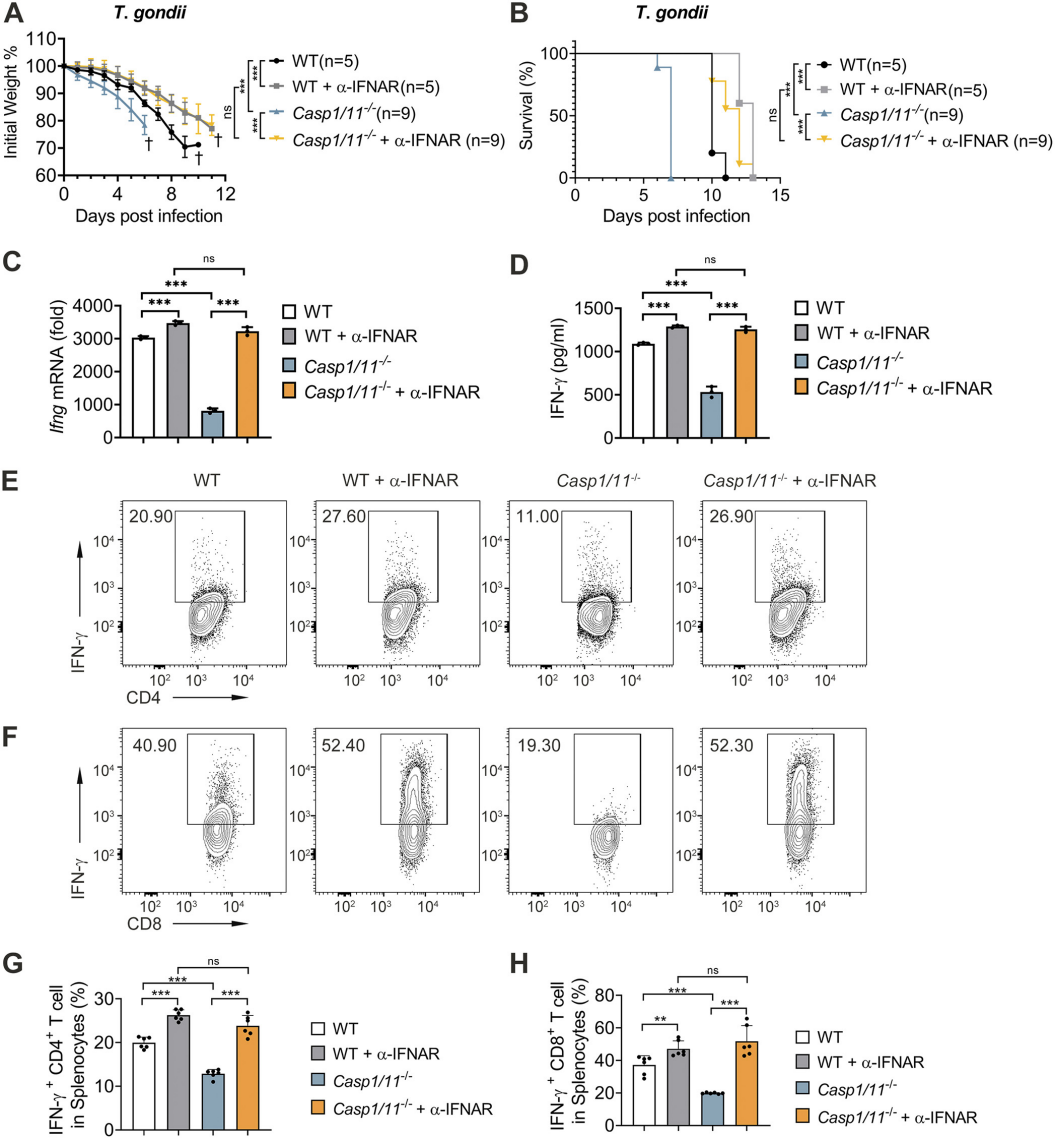
Blockage of type I IFN receptor reverses the susceptible phenomenon and IFN-γ response in *Casp1/11^−/−^* mice *in vivo*. (A and B) WT and *Casp1/11^−/−^* mice (each group with indicated numbers) were intraperitoneally infected with PRU tachyzoites (1 × 10^4^) with anti-IFNAR antibody treatment or isotype control, and daily changes of body weight (A) along with survival rates (B) were recorded. (C to G) WT and *Casp1/11^−/−^* mice (*n* = 3 for each group) were intraperitoneally infected with PRU tachyzoites (1 × 10^5^) with or without anti-IFNAR1 antibody treatment, and *Ifnb* mRNA expression in splenocytes (C) or IFN-β secreted in sera (D) were measured at day 5 postinfection. Splenocytes collected on day 5 postinfection were subjected to the test of percentages of IFN-γ^+^CD4^+^ T cells and IFN-γ^+^CD8^+^ T cells using flow cytometry and representative contour plots (E and F) along with statistical graphs (G and H) are shown. Data are representative of three independent experiments and plotted as mean ± SD. *, *P < *0.05; **, *P < *0.01; ***, *P < *0.001 versus corresponding control. Dagger denotes mouse death. See also Fig. S5.

Inflammasome has been reported to protect the host during T. gondii infection through multiple mechanisms (22, 41, 42). We sought to determine whether inflammasome and IL-1β strengthen anti-T. gondii immune responses by relying on suppressing type I IFN. To this end, we blocked IFN-I/IFNAR axis by using anti-IFNAR antibodies in PRU infected WT or *Casp1/11*^−/−^ mice and found blockage of IFNAR in WT or caspase-1/11-deficient mice showed slower weight loss, longer survival time, and lower SNAP scores compared to the isotype control group, and there were no differences between WT and *Casp*1/11^−/−^ mice when IFNAR were blocked (Fig. 6A and B, Fig. S5). These results suggest that inflammasome promotes anti-T. gondii immune responses by suppressing IFN-I signaling. Moreover, we also measured IFN-γ expression in the splenocytes (Fig. 6C) and serum (Fig. 6D), as well as its production by T cells (Fig. 6E to H) of WT or *Casp1/11*^−/−^ mice with or without anti-IFNAR antibodies treatment post-PRU infection. We discovered that anti-IFNAR antibodies treatment enhanced IFN-γ production in PRU-infected WT or *Casp1/11*^−/−^ mice, which was consistent with the phenomenon in IFNAR deficient mice. Furthermore, the differences in IFN-γ production between WT and *Casp1/11*^−/−^ mice were abolished when mice were treated with anti-IFNAR antibodies (Fig. 6C to H). These findings suggest the protective function of the inflammasome in host anti-T. gondii immunity relies on downregulating IFN-I, which enhances IFN-γ production.

10.1128/mbio.02361-22.5 FIG S5 Blockage of type I IFN receptor can reverse the IFN-γ response in *Casp1/11^−/−^* mice in vivo. (Related to Fig. 6) WT and *Casp1/11^−/−^* mice were intraperitoneally infected with PRU tachyzoites (1 × 10^4^) with anti-IFNAR1 antibody treatment or isotype control, and daily SNAP scores were recorded. Data are representative of three independent experiments and plotted as mean ± SD. *, *P < *0.05; **, *P < *0.01; ***, *P < *0.001 versus corresponding control. Dagger denotes mouse death. Download FIG S5, TIF file, 0.1 MB. Copyright © 2022 Hu et al. 2022 Hu et al. https://creativecommons.org/licenses/by/4.0/ This content is distributed under the terms of the Creative Commons Attribution 4.0 International license.

### SOCS1 induced by IL-1β signaling inhibits the TBK1-IRF3 signaling pathway.

**FIG 7:**
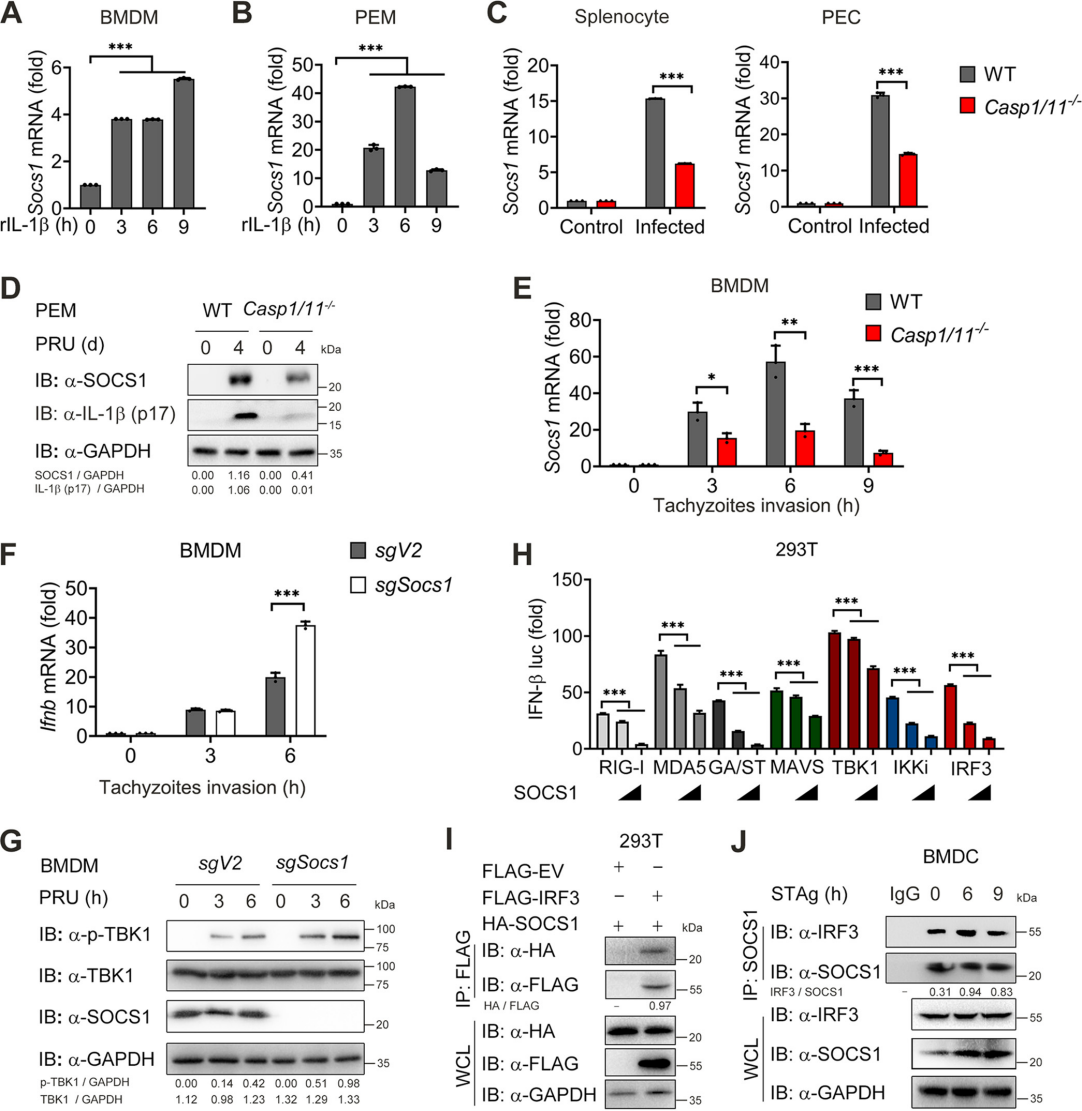
SOCS1 induced by IL-1β inhibits the TBK1-IRF3 signaling pathway. (A and B) WT BMDM (A) or PEM (B) were stimulated with recombinant mouse IL-1β (2 μg/mL) for indicated times, and RNA from the macrophages was isolated to detect expression of *Socs1* mRNA levels using qRT-PCR. (C and D) WT and *Casp1/11^−/−^* mice (*n* = 3 for each group) were intraperitoneally infected with T. gondii (1 × 10^5^ PRU tachyzoites), and splenocytes along with PECs were collected on day 4 postinfection to detect the expression of *Socs1* mRNA levels using qRT-PCR (C). PEM was subjected to test SOCS1 and IL-1β expression using Western blot analysis (D). (E) WT and *Casp1/11^−/−^* BMDM were infected with PRU tachyzoites at MOI = 3 for 0, 3 and 6 h, then subjected to detect expression of *Socs1* mRNA levels using qRT-PCR. (F and G) WT and SOCS1 KO BMDM were infected with PRU tachyzoites at MOI = 3 for 0, 3 and 6 h, then subjected to detect expression of *Ifnb* mRNA levels using qRT-PCR (F) along with expression of pTBK1, TBK1, and SOCS1 using Western blot analysis (G). (H) Dose responses of luciferase signals from 293T cells after cotransfection of the indicated plasmids with different amounts of plasmid (0, 300, and 600 ng) encoding *Socs1*. (I) 293T cells were cotransfected with vectors expressing Flag-IRF3 or Flag-EV and HA-SOCS1 for 24 h. Cellular lysates were subjected to an immunoprecipitation assay with Flag beads and Western blotting with the indicated antibodies. (J) Protein lysates of BMDC stimulated with STAg for indicated time points (above lanes) were subjected to immunoprecipitation with anti-SOCS1 antibody and immunoblot analysis with indicated antibodies. Graphs show the mean and SEM of three independent experiments. *, *P < *0.05; **, *P < *0.01; ***, *P < *0.001 versus corresponding control. NS, not significant. See also Fig. S6.

To further elucidate the mechanisms by which ablation of inflammasome signaling promotes IFN-I signaling activation during toxoplasmosis, we examined the mRNA expression of several potential negative regulators, such as *Rtp4*, *Fosl1*, *A20*, *Rnf5*, *Nlrc3*, and *Duba* (52–58), in PEMs treated with recombinant IL-1β, and observed mRNA levels of *Socs1*, *Rtp4*, *Fosl1*, and *A20* increased in IL-1β treated PEMs and BMDMs at indicated times (Fig. 7A and B, Fig. S6A to F). Next, we detected expression patterns of *Socs1*, *Rtp4*, *Fosl1*, and *A2*0 mRNA in freshly isolated splenocytes from PRU-infected WT and *Casp1/11^−/−^* mice. Strikingly, only the mRNA of *Socs1* was markedly decreased in splenocytes and PECs of *Casp1/11^−/−^* mice compared with WT mice (Fig. 7C). In contrast, we did not observe appreciable changes in the expression of *Rtp4*, *Fosl1*, and *A2*0 between PRU-infected WT and *Casp1/11^−/−^* mice (Fig. S6G). Meanwhile, SOCS1 protein expression was reduced in PECs isolated from *Casp1/11^−/−^* mice on day 4 after PRU infection compared to WT mice (Fig. 7D). To further confirm these observations, we infected BMDMs using PRU tachyzoites and checked mRNA level of *Socs1* in BMDMs. Consistent with data *in vivo*, loss of caspase-1/11 impaired generation of SOCS1 during PRU infection *in vitro* (Fig. 7E). Together, these findings indicate that SOCS1 can be induced by inflammasome-coupled IL-1 signaling after T. gondii infection, and potentially negatively regulates IFN-I signaling.

10.1128/mbio.02361-22.6 FIG S6 The expression of other negative regulated molecules in vivo and in vitro. (Related to Fig. 7) (A to G) mRNA levels for *Rtp4* (A), *Fosl1* (B), *A20* (C), *Rnf5* (D), *Nlrc3* (E), *Duba* (F) in WT PEM after stimulation with mouse rIL-1β (2 μg/mL) for indicated time points by qRT-PCR. (G) mRNA levels for *Rtp4*, *Fosl1*, and *A20* in the splenocytes at 4 days postinfection from WT and *Casp1/11^−/−^* mice infected with T. gondii (1 × 10^5^ PRU tachyzoites). Data are representative of three independent experiments and plotted as mean ± SD. *, *P < *0.05; **, *P < *0.01; ***, *P < *0.001 versus corresponding control. Download FIG S6, TIF file, 0.4 MB. Copyright © 2022 Hu et al. 2022 Hu et al. https://creativecommons.org/licenses/by/4.0/ This content is distributed under the terms of the Creative Commons Attribution 4.0 International license.

To further determine the role of SOCS1 in limiting IFN-I expression, we genetically ablated the Socs1 gene in BMDMs using the CRISPR/Cas9 system. PRU infection led to higher levels of IFN-I production and phosphorylation of TBK1 in SOCS1-deficient BMDMs (Fig. 7F and G). Because SOCS1 is a negative regulator which can inhibit type I IFN and NF-κB signaling by interacting with MyD88, IRAK1, or STAT1 (59–61), we next sought to explore the molecular target of SOCS1 in PRU-induced type I IFN signaling. We transfected 293T cells with IFN-β luciferase reporter vector with downstream adaptors and SOCS1 plasmids and found that IFN-β-Luc activity was strongly activated by overexpression of RIG-I, MDA5, MAVS, cGAS/STING (GA/ST), TBK1, IKKi, or IRF3, but all of these activities were inhibited when SOCS1 was cotransfected at increasing concentrations (Fig. 7H), suggesting that it may block IFN-β activation at the signaling level of IRF3. To test this prediction, we transfected 293T cells with HA-tagged SOCS1 and Flag-tagged IRF3. Coimmunoprecipitation (co-IP) assay revealed that SOCS1 interacted with IRF3 (Fig. 7I). Meanwhile, we found the endogenous interaction between SOCS1 and IRF3 during PRU STAg stimulation (Fig. 7J). These results indicate that inflammasome-coupled IL-1 signaling induces the production of SOCS1, suppressing IFN-I signaling by targeting IRF3.

## DISCUSSION

**FIG 8:**
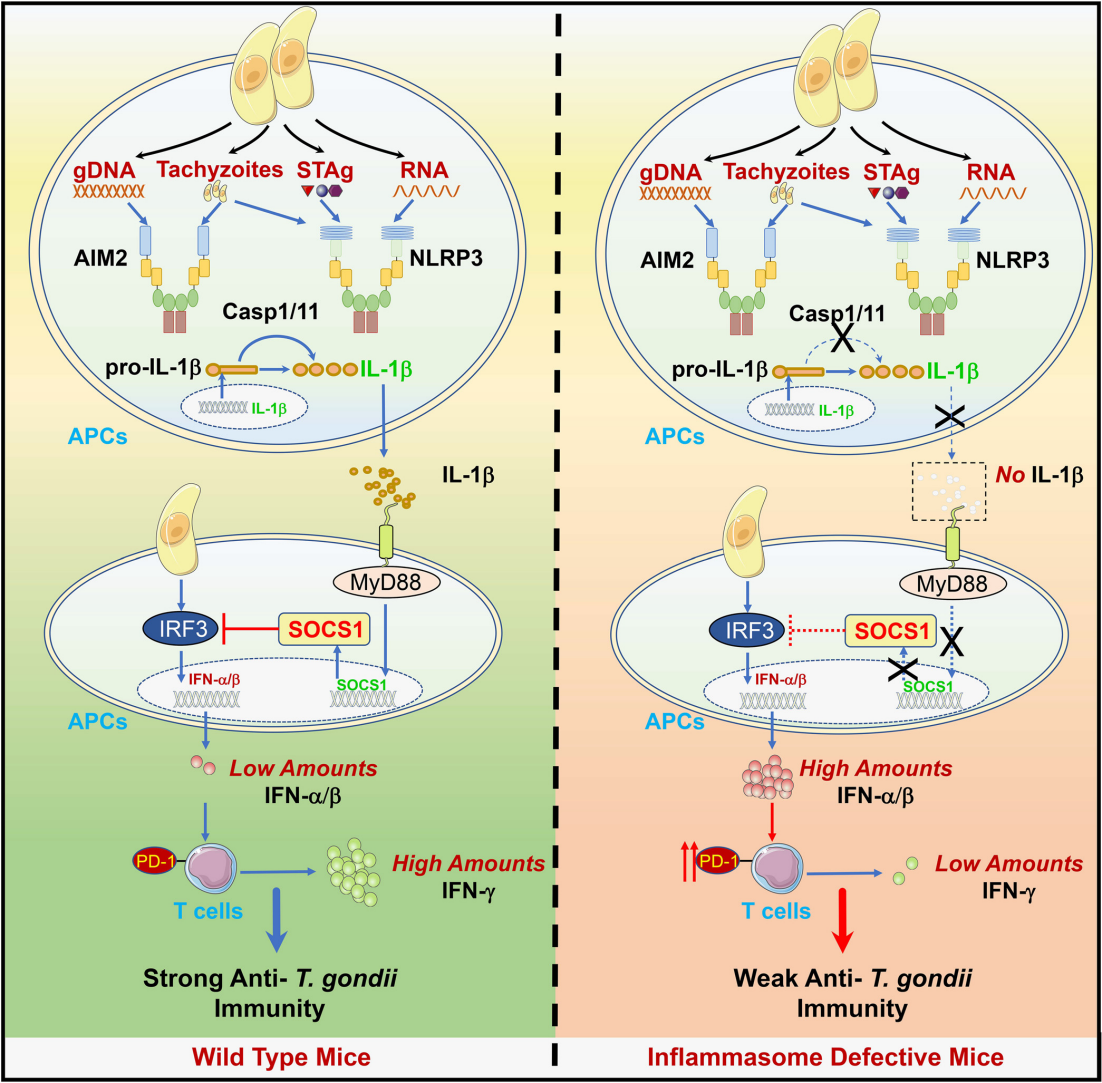
A schematic model for this study. A schematic model shows that the infection of T. gondii, PRU, triggers the activation of inflammasome signaling and IL-1β production through the interaction between PRU-associated PAMPs and host inflammasomes. Briefly, PRU tachyzoites can both activate NLRP3 and AIM2, while PRU STAg and RNA only act on NLRP3. Besides, PRU gDNA is only sensed by AIM2. Followingly, the IL-1β-IL-1R axis induces an increased expression of SOCS1, which inhibits excitation of IFN-I that induces PD-1 expression in T cells via interacting with IRF3, thus preventing the overproduction of IFN-I and confirming a normal production of IFN-γ to protect the host. However, IL-1β secretion is abolished in the host inflammasomes or caspase1/11 is deficient, and expression of SOCS1 is no longer upregulated to suppress IFN-I signaling, and the host dies due to large amounts of IFN-β.

Although inflammasomes have been reported vital for defending against T. gondii infection (41, 62), little is known about the underlying mechanism. In this study, we demonstrated that the mice deficient in AIM2, NLRP3, or caspase-1/11 were more susceptible to the lethal *Toxoplasma* PRU strain infection, suggesting their roles in sensing T. gondii PAMPs and triggering IL-1β production. Besides, we also showed that *Ifnar*^−/−^ mice were partially resistant to this parasite infection, identifying the harmful role of IFN-I in host anti-T. gondii immunity. Mechanistically, our results indicated that gDNA and tachyzoites of T. gondii were preferentially recognized by AIM2, while STAg, RNA, and tachyzoites were sensed by NLRP3. The activations of AIM2 and NLRP3 led to the cleavage of pro-IL-1β and release of IL-1β, which couples with IL-1R and induces the production of negative regulator SOCS1. The induction of SOCS1 then inhibited TBK1-IRF3-dependent IFN-I signaling by interacting with IRF3, resulting in small amounts of IFN-I production, which protected the host from T. gondii infection. In contrast, SOCS1 expression was markedly reduced in the inflammasome-defective host, activating TBK1-IRF3 mediated type I IFN signaling and making the host more susceptible to T. gondii infection. Thus, our study highlights the cross talk between inflammasome and IFN-I signaling during T. gondii infection, in which IL-1 activation negatively regulates IFN-I signaling by promoting the expression of SOCS1, which is a negative regulator that targets IRF3 (Fig. 8).

NLRC1, NLRC4, NLRP3, and AIM2 are the major inflammasome components, but these sensors' ligands and roles are distinct during different pathogen infections. A recent report suggested that T. gondii-induced NLRP3 inflammasome activation was strongly associated with the phosphorylation of p38 MAPK (63). Besides, there was evidence that T. gondii could induce an atypical apoptosis pathway involving the AIM2 and promotes apoptosis via ASC and caspase-8 (27). However, direct evidence is insufficient to support how inflammasome sensors are activated or their specific function in the course of T. gondii infection. In this study, we demonstrated that nucleic acids secreted from T. gondii, including gDNA and RNA, could be directly sensed by different inflammasome sensors. We found the activation of AIM2-mediated inflammasome by gDNA while toxoplasma RNA mainly activated NLRP3 in murine primary macrophages and dendritic cells (DCs) during T. gondii infection *in vitro*, which could help us strengthen the knowledge of innate immune responses during the T. gondii PRU infection.

Besides inflammasomes, type I IFN acts as another pivotal cytokine in innate immunity against pathogen infections, and the cross talk between them has been extensively investigated during other pathogen infections. Previous studies showed that IFN-I could inhibit NLRP1- and NLRP3-activated inflammasomes by STAT1 (64). Although type I IFN was found to limit pro-IL-1β production via inducing IL-10 signals through STAT3 (64, 65), evidence also supported the importance of type I IFN in triggering the activation of the inflammasome. Recent studies have indicated that IFN-I can induce caspase-11 to activate the noncanonical inflammasome pathway and pyroptosis via ROS and/or the Cpb1-C3-C3aR complement pathway and can also promote the activation of AIM2 or NLRP3 and the cell death during Francisella novicida or influenza A virus infection (66–69). Conversely, inflammasome activation can also dampen IFN-I signaling. For instance, inflammasome activation triggered caspase-1-mediated cleavage of cGAS to impair IFN-I responses to DNA virus and Mycobacteria bovis infection (70, 71). Inflammasome-mediated antagonism of type I IFN was also demonstrated to enhance Rickettsia pathogenesis (72). Additionally, our previous study demonstrated that the inflammasome could negatively regulate MyD88-IRF7 type I IFN signaling and anti-malaria immunity (47). Here, we found that this cross talk also existed during T. gondii infection, in which the activation of the inflammasome suppressed IFN-I signaling and production of IFN-β, which highlights, for the first time, the cross talk between these two innate signaling pathways in anti-T. gondii immunity.

As the target of inflammasome signaling, the role of type I IFN during T. gondii infection is unknown, although it is required for constraining pathogen infection like viruses or *Plasmodium* (28, 47, 59, 73). In this study, we found that mice lacking IFNAR could be partially resistant to T. gondii PRU intraperitoneal infection, which was different from the chronic oral infection, and mice administered with recombinant IFN-I displayed to be more susceptible to this parasite infection, indicating IFN-I is detrimental to the hosts ability to generate anti-T. gondii immune responses. The distinct role of IFN-I during acute and chronic T. gondii is interesting, and the opposing capability of this cytokine has been already well studied in other diseases, such as tumor models and virus infection, including SARS-CoV-2 (74–77). Moreover, one of the negatively regulated functions of IFN-I is to suppress T cell responses during multiple diseases by altering T cell trafficking, inducing T cell apoptosis, inhibiting T cell proliferation, and promoting coinhibitory receptors expression in T cells (78–82). Here, we found IFN-I promoted PD-1, but not LAG3 or CTLA4 expression in both CD4^+^ and CD8^+^ T cells during toxoplasmosis, which was partly consistent with a recent report that IFN-I induces PD-1/TIM-3/LAG-3 while inhibiting TIGIT expression in human T cells (82), but the underlying mechanism needs to be further explored. As a result of upregulated PD-1 expression, IFN-I negatively regulates IFN-γ production in T cells, which was widely considered to protect the host against T. gondii infection (43, 45, 46), thus revealing a novel regulatory mechanism between different IFN-I and IFN-γ.

It is well understood that the innate immune system has developed the capacity to recognize self- and nonself-nucleic acids, which is crucial for IFN-I activation (83–85). To explore the mechanism by which IFN-I signaling is activated during T. gondii infection, we isolated both genomic DNA and RNA from PRU tachyzoites and found that the parasite nucleic acids could trigger IFN-I production *in vitro*, which is consistent with the results obtained from *in vivo* experiments. However, further investigations are required to elucidate how nucleic acids are recognized by host pattern-recognition receptors (PRRs) such as cGAS, RLRs, and TLRs.

Given this, we consider that we have revealed a novel protective mechanism of inflammasomes through negatively regulating harmful overactivation of IFN-I. It is the first report about cross talk between these two signaling pathways during T. gondii infection. Collectively, these findings uncover an interesting cross talk between IFN-I and inflammasome signaling, emphasizing the significance of the double-negative feedback regulative loop in innate immunity against T. gondii and perhaps also other pathogen infections.

During T. gondii infection, increasing negative factors from the pathogen or host have been identified to control IFN-I signaling tightly. A recent study showed that *TgIST*, a T. gondii-secreted effector, bound to STAT1/STAT2 heterodimers and blocked type I IFN signaling (31). In addition, evidence indicated that *TgROP18I* bound to IRF3 and blocked the translocation of IRF3 from the cytosol to the nucleus (35). Distinct from these exogenous regulators from pathogens, here we have identified SOCS1, an endogenous negative regulator, targeted IRF3 and suppressed IFN-I activation. SOCS1 is a classical negative factor suppressing JAK/STAT signaling and NF-κB signaling (60, 61, 86, 87). Previously, we reported that SOCS1 induced by STING-/MAVS-mediated signaling could inhibit MyD88-mediated type I IFN signaling in plasmacytoid dendritic cells (pDCs), and we also verified that inflammasome activation could enhance IL-1β-mediated signaling and upregulate SOCS1, thus inhibiting MyD88-IRF7 mediated IFN-I signaling in pDCs during malaria infection (47, 59). In this study, we further demonstrated that SOCS1 induced by T. gondii triggered inflammasome signaling could restrict IFN-I production by interacting with IRF3 in macrophages and DCs. The role of SOCS1 in *Plasmodium* and T. gondii infection is similar, although its regulation of IFN-I occurred in different cell types. During malaria, AIM2 and NLRP3-induced CASP1-dependent inflammasome signaling induces the release of IL-1β in pDCs and activates IL-1 signaling, which induces negative regulator SOCS1 in a MyD88-TRAF3-IRF3-dependent manner and inhibits MyD88-IRF7-dependent type I IFN signaling in pDCs (47, 59). In contrast, SOCS1 induced by T. gondii infection depends on inflammasome signaling and suppresses TBK1-IRF3 signaling in macrophages and conventional DCs. Genetic deletion of SOCS1 *in vitro* promoted T. gondii-induced IFN-I expression. However, the molecular mechanism by which SOCS1 impacts IRF3-mediated IFN-I remains to be further explored.

The inflammasome can protect the host against T. gondii infection in different ways. Here, we report a novel protective mechanism by which inflammasome signaling attenuates IFN-I production by inducing SOCS1-mediated inhibition on IRF3 in macrophages and define the detrimental function of IFN-I during T. gondii infection. In summary, our findings suggest novel cross talk and its mechanism between the two important weapons with opposite functions of innate immunity during T. gondii infection, which highlight the complex relationship between host and T. gondii infection and may offer potential therapeutic targets for the development of safe and effective toxoplasmosis vaccines.

## MATERIALS AND METHODS

### Toxoplasma gondii and its infection.

The parasite used in this research was the type II PRU strain, and we also employed PRU with a red fluorescent protein (RFP) expression for parasite load measurement. Parasites were propagated intracellularly in rat embryonic fibroblasts (ATCC). Tachyzoites were isolated and purified through a 5.0-μm Nuclepore membrane and washed twice with PBS. For immunological index, like cytokine or cell population assay, mice were intraperitoneally injected with 1 × 10^5^ PRU tachyzoites suspended in PBS. For survival, weight change, SNAP, and parasite burden assay, mice were intraperitoneally injected with 1 × 10^4^ PRU tachyzoites suspended in PBS.

### Animals.

C57BL/6 mice, aged 6 to 8 weeks old, were purchased from the Experimental Animal Centre of Southern Medical University. *Aim2^−/−^* mice were kindly gifted by Shuo Yang (Nanjing Medical University, China). *Nlrp3^−/−^* and *Casp1/11^−/−^* mice were from Zi Li (Guangzhou Medical University, China). *Ifnar^−/−^* mice were purchased from The Jackson Laboratory. All mouse-related procedures were performed according to experimental protocols authorized by the Southern Medical University Animal Care and Use Committee (SMUL2019243).

### SNAP score and spleen index analysis.

T. gondii PRU-infected mice were monitored daily for the development of neuropathology using a 12-point clinical scoring system that rated mice from a score of 0 (no abnormalities) to 12 (moribund) as previously described and improved from the simple neuro assessment of asymmetric impairment (SNAP) scoring system (44). Briefly, animals were judged by testing five indexes: interactions/reflex, cage grasp, visual placing, gait/posture/appearance, and capacity to hold their body weight on a baton. Each category was scored from 0 to 3, where 0, 1, 2, and 3 represented normal individuals, intermediate, no ability, and moribund, respectively, to describe the parameter (88). Spleen indices were calculated according to the following formula: spleen index = weight of thymus or spleen (mg)/body weight (g), as described previously (89).

### Parasite load measurement.

Parasite load *in vivo* was measured using bioluminescence imaging. Infected wild-type and indicated transgenic mice were imaged 5 days postinfection to track the parasite burden *in vivo*. Mice were imaged with the In-Vivo Fx Pro imaging system (Bruker) with continuous administration of 2.5% isoflurane via nose cone. Images were analyzed using Bruker MISE software. For parasite growth detection in L929 cells, PRU with an RFP expression was added into indicated variously treated cells, and the parasites’ burden was determined by both red fluorescence intensity and the transcriptive levels of *ITS-1* region conserved in all T. gondii strains (90).

### Primary cells culture.

Bone marrow cells were isolated from the tibia and femur of indicated mice. BMDMs were cultured in Dulbecco’s modification of Eagle’s medium (DMEM) medium with 10% FBS, 1% penicillin-streptomycin, and 10% L929 conditioned media containing macrophage-colony-stimulating factor (M-CSF) for 6 days. BMDCs were cultured in Roswell Park Memorial Institute (RPMI) 1640 medium with 10% FBS, 1% penicillin-streptomycin, 55 μM β-mercaptoethanol, 20 ng/mL murine GM-CSF, and 10 ng/mL murine IL-4 for 6 days.

### Isolation of Toxoplasma gondii gDNA, RNA, and STAg and cell stimulation.

Toxoplasma gondii gDNA, RNA, and STAg were isolated. First, a 0.5 μm filter was used to avoid the influence of cell debris. Parasites were collected after filtration through centrifugation at 2,000 *g* for 10 min, and the lysate was incubated with buffer A (150 mM NaCl, 25 mM EDTA, 10% SDS, and protein kinase) overnight. gDNAs were isolated using phenol-chloroform extraction, and RNAs were isolated using TRIzol reagent (Invitrogen). As for STAg, the lysate was suspended in PBS, and repetitive freeze-thawing at 37°C and −196°C five times was performed to ensure the complete release of the *Toxoplasma* antigens. Then the mixture was centrifuged at 4°C, 12,000 *g* for 30 min. The supernatant was transferred to another new microcentrifuge tube and stored at −80°C. For gDNA, RNA, and STAg stimulation in cells, depurated T. gondii gDNA and RNA were transfected into cells through StarFect high-efficiency transfection reagent (GenStar) at the indicated time. For T. gondii infection *in vitro*, purified tachyzoites were administered at a multiplicity of infection (MOI) of 3.

### Enzyme-linked immunosorbent assay (ELISA).

The cell supernatants and serum of mice were detected using mouse IFN-β (R&D systems), IFN-γ (eBioscience), and IL-1β (eBioscience) ELISA kits following the manufacturer’s instructions. Absorbance was measured at 450 nm by the Multiscan FC (Thermo Fisher).

### RNA preparation and Qpcr.

Total RNA was acquired from the spleen, lymph node, or stimulated cells through the TRIzol reagent (Invitrogen). The complementary cDNA was generated using the Starscript II first-stand cDNA synthesis kit (GenStar). Realtime PCR was performed on QuantStudio 6 flex (Thermo Fisher) using RealStar green power mixture (GenStar) with primers. The sequences of primers are shown in Table S1.

10.1128/mbio.02361-22.7 TABLE S1 Primers sequences for quantitative RT-PCR. Download Table S1, DOCX file, 0.02 MB. Copyright © 2022 Hu et al. 2022 Hu et al. https://creativecommons.org/licenses/by/4.0/ This content is distributed under the terms of the Creative Commons Attribution 4.0 International license.

### Immunoblot analyze.

Whole-cell extracts were prepared after invasion by PRU tachyzoites or stimulation by its associated PAMPs. Supernatants were precipitated with methanol/chloroform, boiled for 10 min with SDS loading buffer (Cell Signaling Technology), and resolved on SDS-PAGE gels. Proteins were transferred to polyvinylidene difluoride (PVDF) membranes (Millipore) and incubated with the appropriate antibodies. Immobilon Western Chemiluminescent HRP Substrate (Millipore) was used for protein detection. The antibodies used are shown in Table S2.

10.1128/mbio.02361-22.8 TABLE S2 Reagents and antibodies used in this study. Download Table S2, DOCX file, 0.02 MB. Copyright © 2022 Hu et al. 2022 Hu et al. https://creativecommons.org/licenses/by/4.0/ This content is distributed under the terms of the Creative Commons Attribution 4.0 International license.

### Immunoprecipitation.

For endogenous immunoprecipitation, whole-cell lysates were prepared after stimulation with STAg, followed by incubation overnight with the anti-SOCS1, then plus-agarose A/G beads (Pierce) were incubated the next day for 4 h. Beads were then washed five times with low-salt lysis buffer (50 mM HEPES, 150 mM NaCl, 1 mM EDTA, 10% glycerol, 1.5 mM MgCl_2_, and 1% Triton X-100), and immunoprecipitates were eluted with 2×SDS loading buffer and resolved by SDS-PAGE. For exogenous immunoprecipitation, HEK293 T cells plated in 24-wells were cotransfected with vectors expressing Flag-IRF3 or Flag-EV and HA-SOCS1 for 24 h. Cellular lysates were subjected to an immunoprecipitation assay with Flag beads overnight. Beads were then washed 5 times with low-salt lysis buffer (50 mM HEPES, 150 mM NaCl, 1 mM EDTA, 10% glycerol, 1.5 mM MgCl_2_, and 1% Triton X-100), and immunoprecipitates were eluted with 2×SDS loading buffer and resolved by SDS-PAGE.

### Flow cytometry and intracellular staining.

For intracellular IFN-γ staining, splenocytes from indicated mice were cultured in the medium containing Cell Stimulation Cocktail (plus protein transport inhibitors) (Invitrogen) for 12 h, then incubated with anti-CD4 and anti-CD8a for surface staining. After fixation and permeabilization (BD; Fix & Perm), splenocytes were stained against anti-IFN-γ. All samples were operated on BD LSRFortessa cytometer (BD sciences). The data were analyzed via FlowJo X software (Tree Star).

### Luciferase and reporter assays.

HEK293T cells were plated in 24-well plates and transfected with plasmids encoding the IFNB luciferase reporter (firefly luciferase; 200 ng) and Prl-TK (Renilla luciferase; 50 ng), together with different plasmids. After 24 h, the cells were lyzed with indicated buffer, and luciferase activity was measured with a dual-luciferase assay (Promega, E1910) with a Luminoskan ascent luminometer (Thermo Fisher Scientific). Reporter gene activity was determined by normalization of the firefly luciferase activity to Renilla luciferase activity. The values were means ± SD of 3 independent transfections performed in parallel.

### Generation of SOCS1 knockout cell lines.

Target sequences (SOCS1 guide: 5′-CACCGGATGCGCCGGTAATCGGAGT-3′) were cloned into pLenti-CRISPR-v2 by cutting with *Bsm*BI to generate SOCS1 knockout (KO) cells. The lentiviral vectors were transfected with the expression plasmid for the vesicular stomatitis virus G protein into the HEK293T cells for the preparation of the lentivirus. The medium was refreshed the next day and the supernatant containing lentivirus was collected 48 h after transfection, filtered through a 0.45 μm filter, and subsequently used to infect cells with Polybrene (8 μg/mL). Next, BMDMs were infected by incubation with lentivirus-containing supernatant for 12 h and subjected to the indicated experiments.

### *In vivo* recombinant IFN-I and anti-IFNAR antibody treatment.

WT and *Casp1/11^−/−^* mice were intraperitoneally infected with T. gondii (indicated PRU tachyzoites). Recombinant IFN-I (IFN-β, 200 ng/kg, R&D systems) was administered by intravenous injection to mice on days 1, 3, and 5 postinfection. Antimouse IFNAR blocking antibody was dissolved in PBS and injected into WT and *Casp1/11^−/−^* mice before infection and at day 2 postinfection in the amount of 500 μg per mouse.

### Quantification and statistical analysis.

Data are subjected to statistical analysis using GraphPad Prism version 8.0 (GraphPad Software) and presented as mean ± SD, unless otherwise stated. *P* values less than 0.05 were considered statistically significant for all data sets. Statistical significance between the two groups was assessed by unpaired *t*-tests.
