# Supplementary figures and images for: Inflammasome Activation Dampens Type I IFN Signaling to Strengthen Anti-Toxoplasma Immunity
Source: mBio. 2022 Oct 10;13(6):e02361-22. doi: 10.1128/mbio.02361-22 (PMC9765454; doi:10.1128/mbio.02361-22)

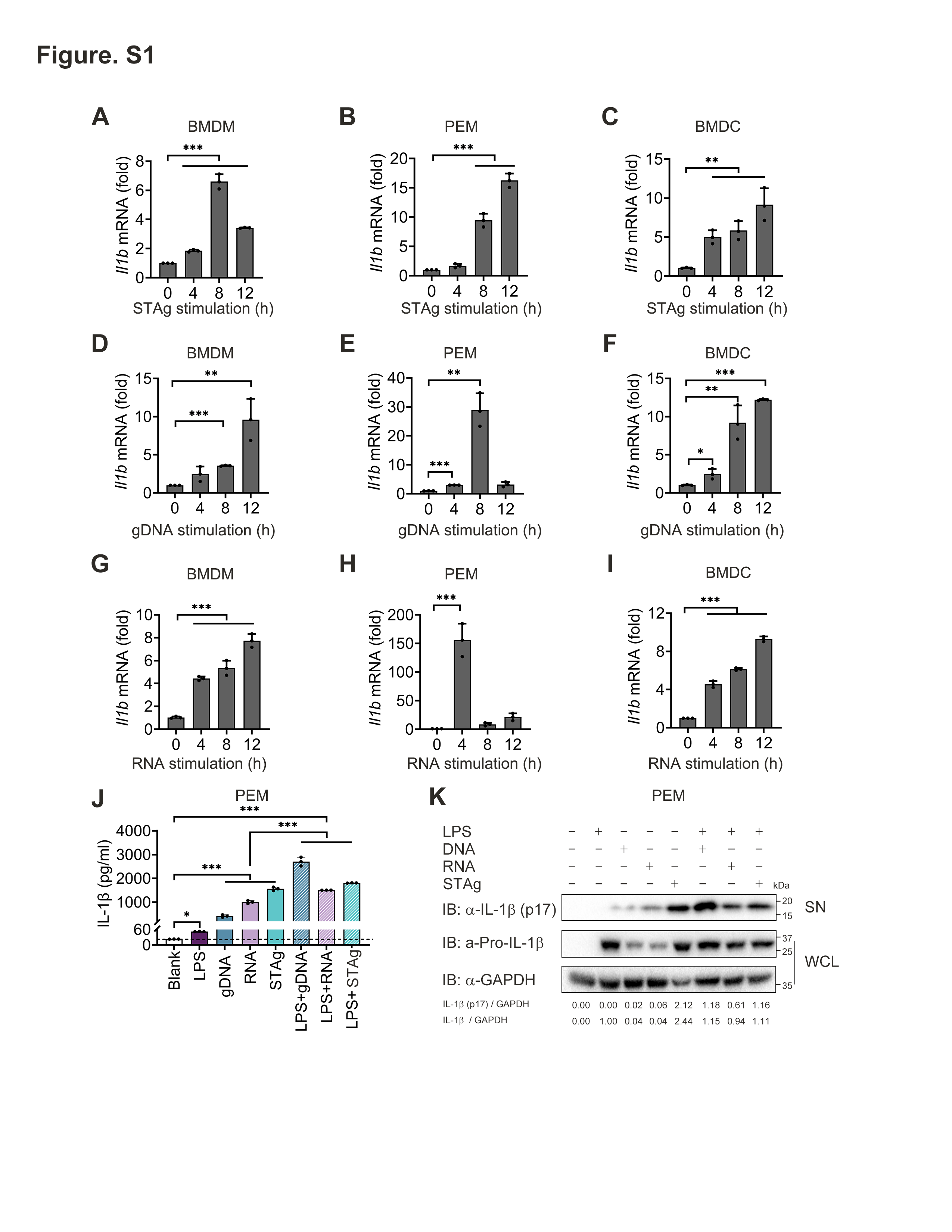

Supplement: FIG S1 [file mbio.02361-22-s0001.tif]

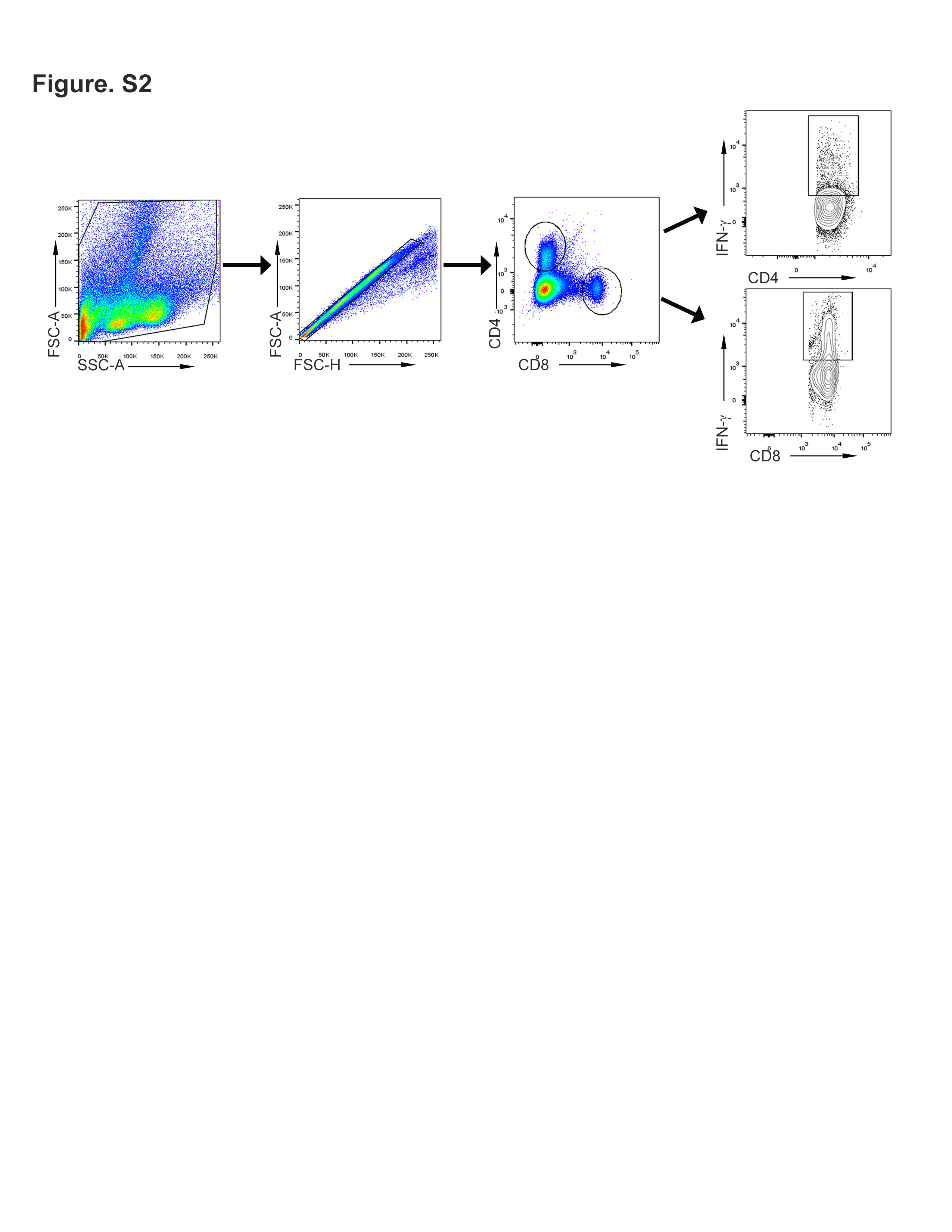

Supplement: FIG S2 [file mbio.02361-22-s0002.tif]

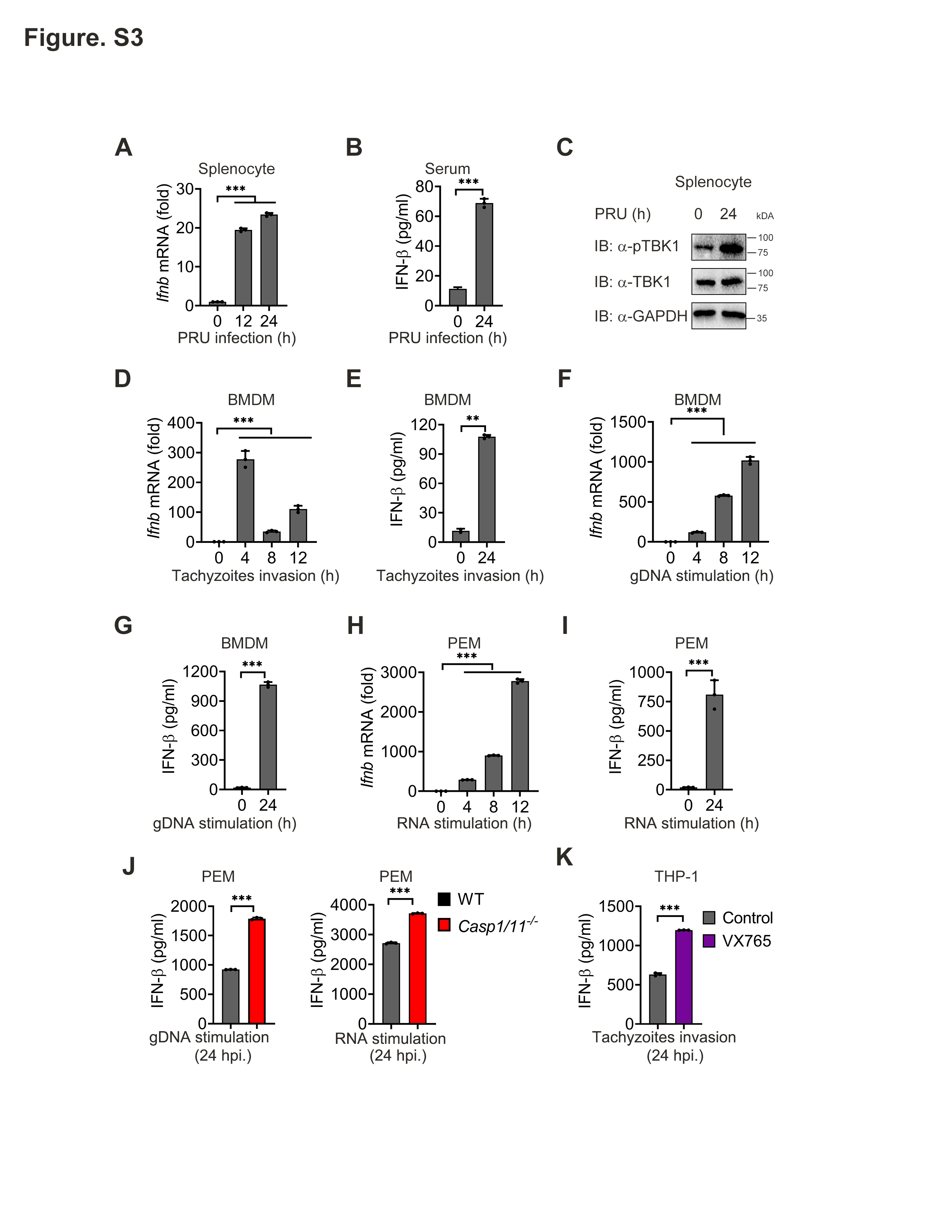

Supplement: FIG S3 [file mbio.02361-22-s0003.tif]

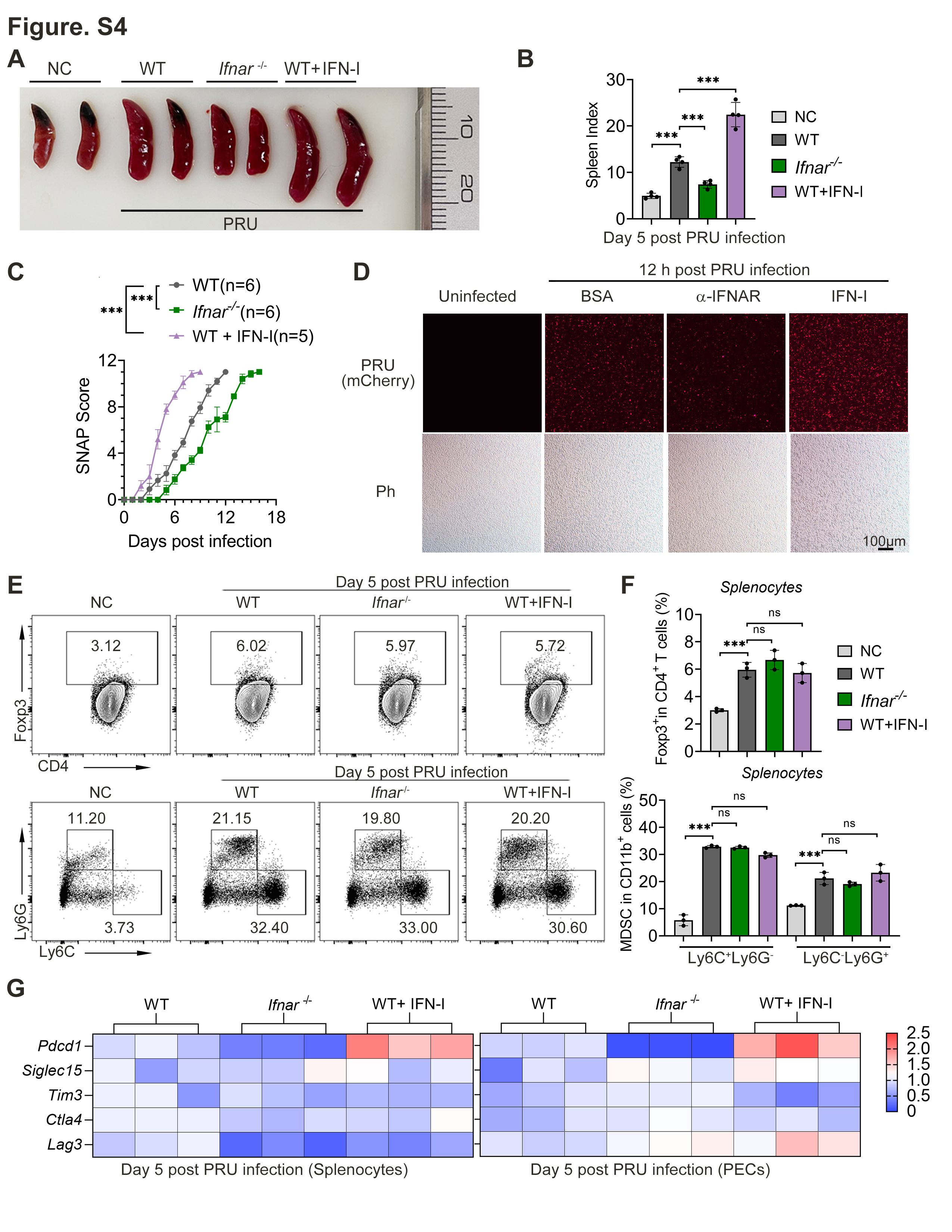

Supplement: FIG S4 [file mbio.02361-22-s0004.tif]

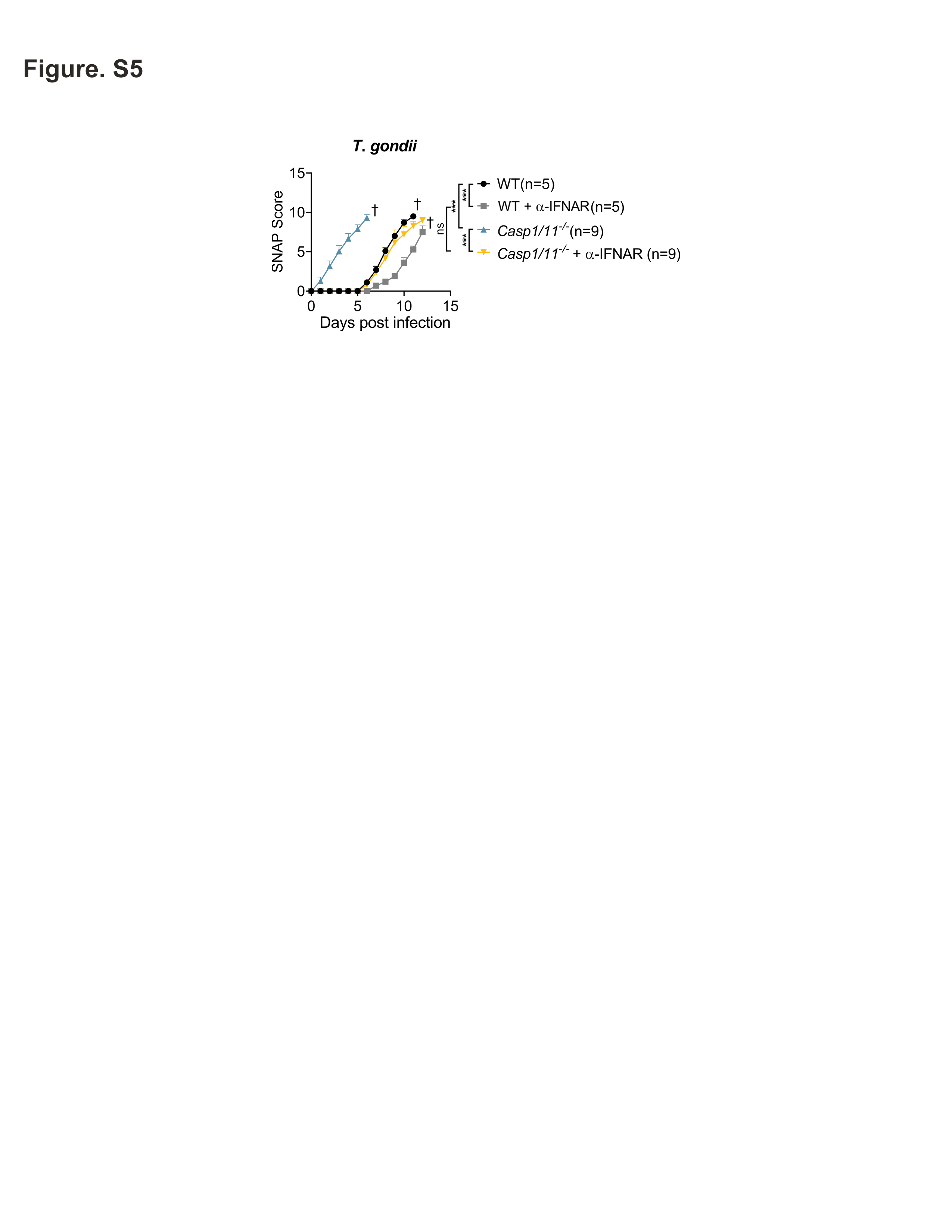

Supplement: FIG S5 [file mbio.02361-22-s0005.tif]

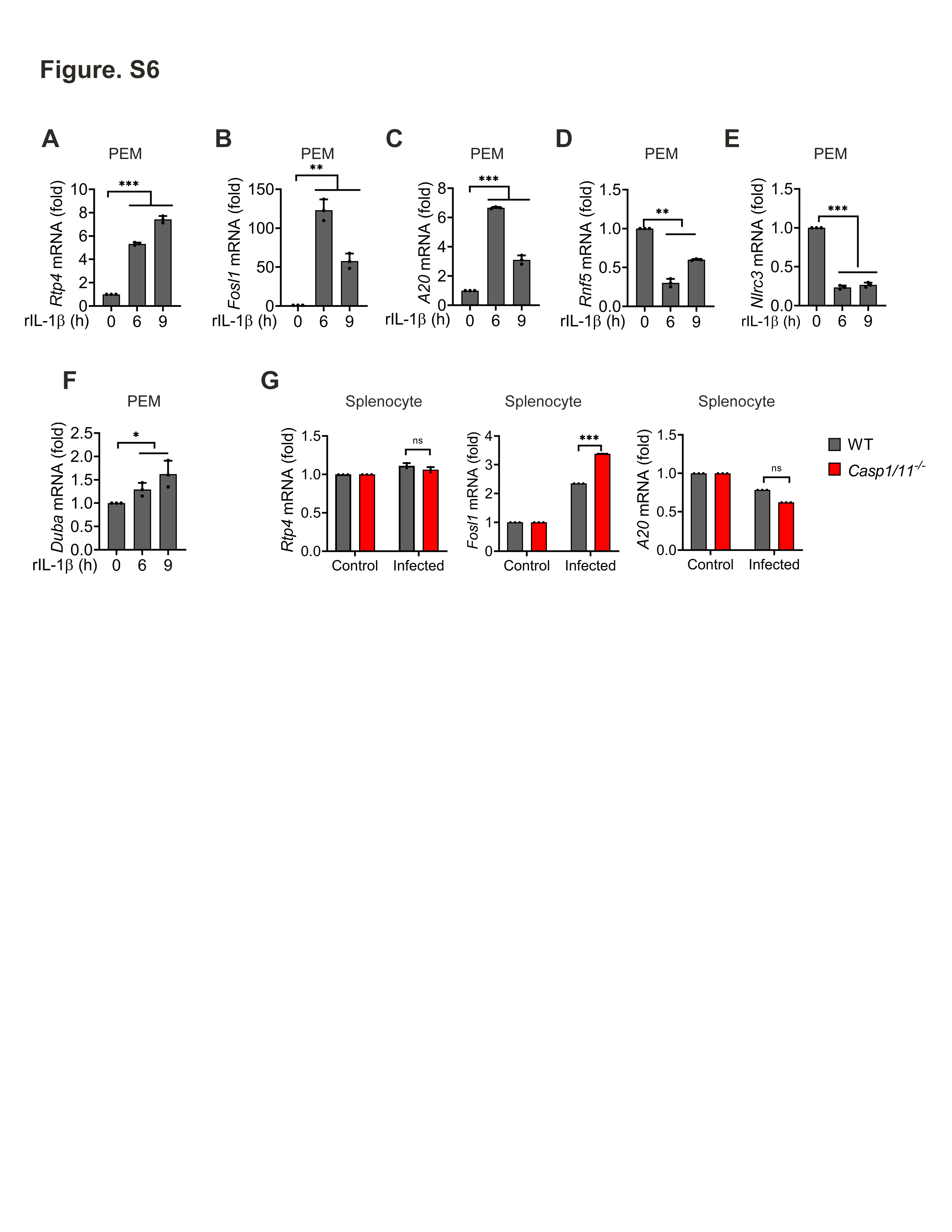

Supplement: FIG S6 [file mbio.02361-22-s0006.tif]
