# Supplementary material for: Inflammasome Activation Dampens Type I IFN Signaling to Strengthen Anti-Toxoplasma Immunity
Source: mBio. 2022 Oct 10;13(6):e02361-22. doi: 10.1128/mbio.02361-22 (PMC9765454; doi:10.1128/mbio.02361-22)
Supplement: TABLE S1 [file mbio.02361-22-s0007.docx]

**Table. S1. Primers sequences for quantitative RT-PCR.**

| **Gene** | **Forward Primer Sequence** | **Reverse Primer Sequence** |
| --- | --- | --- |
| *Il1b* | CACAGCAGCACATCAACAAG | GTGCTCATGTCCTCATCCTG |
| *Ifnb* | TCACCTACAGGGCGGACTTC | TCTCTGCTCGGACCACCATC |
| *Socs1* | CTGCGGCTTCTATTGGGGAC | AAAAGGCAGTCGAAGGTCTCG |
| *Rtp4* | TCCACACCTCTGAGAATGCTG | CATCTGGAACACTGGAACCTGC |
| *Fosl1* | ATGTACCGAGACTACGGGGAA | CTGCTGCTGTCGATGCTTG |
| *A20* | CTCGGAACTTTAAATTCCGC | GGGTAAGTTAGCTTCATCC |
| *Nlrc3* | CAGATTGGTAACAAAGGAGCCA | CGTTCGGTTTATCTTCAGAGCA |
| *Duba* | CAGTGAAGACGAGTATGAAGCTG | AGCCCGAAATAGACAGGCAC |
| *Rnf5* | CAAGAATGCCCGGTGTGTAAA | GGGTGGAGTTTTCAATCTGGGA |
| *Tim3* | TCAGGTCTTACCCTCAACTGTG | GGGCAGATAGGCATTTTTACCA |
| *Ctla4* | TTTTGTAGCCCTGCTCACTCT | CTGAAGGTTGGGTCACCTGTA |
| *Siglec15* | ACACCCCTCCACGGTCTC | CTCACAGCAAACACGTGGCA |
| *Lag3* | CTGGGACTGCTTTGGGAAG | GGTTGATGTTGCCAGATAACCC |
| *Pdcd1* | ACCCTGGTCATTCACTTGGG | CATTTGCTCCCTCTGACACTG |
| *ITS-1* | AATATTGGAAGCCAGTGCAGG | CAATCTTTCACTCTCTCTCAA |
| *Gapdh* | AAGGTCATCCCAGAGCTGAA | CTGCTTCACCACCTTCTTGA |
