# Supplementary material for: Inflammasome Activation Dampens Type I IFN Signaling to Strengthen Anti-Toxoplasma Immunity
Source: mBio. 2022 Oct 10;13(6):e02361-22. doi: 10.1128/mbio.02361-22 (PMC9765454; doi:10.1128/mbio.02361-22)
Supplement: TABLE S2 [file mbio.02361-22-s0008.docx]

**Table. S2. Reagents and antibodies used in this study.**

| **Antibody Name** | **Source** | **Catalog number** |
| --- | --- | --- |
| SOCS1 (A156) | Cell Signaling Technology | #4536 |
| phosphor-TBK1/NAK (Ser^172^) | Cell Signaling Technology | #5483 |
| TBK1/NAK | Cell Signaling Technology | #3013 |
| TBK1(A-6) | Santa Cruz | #11621 |
| IRF3 | Protein Technology | #11312-1-AP |
| IL-1β Rabbit mAb | Protein Technology | #26048-1-AP |
| Cleaved IL-1β (Asp117) (E7V2A) Rabbit mAb (Mouse Specific) | Cell Signaling Technology | #63124 |
| IL-1β (D3H1Z) Rabbit mAb | Cell Signaling Technology | #12507 |
| Caspase-1 (D7F10) | Cell Signaling Technology | #3866 |
| Cleaved Caspase-1 (Asp296) (E2G2I) | Cell Signaling Technology | #89332 |
| β-tubulin | Cell Signaling Technology | #RM2003 |
| GAPDH | Cell Signaling Technology | #RM2002 |
| goat anti-Mouse IgG (H+L)-HRP | Cell Signaling Technology | #RM3001 |
| goat anti-Rabbit IgG (H+L)-HRP | Cell Signaling Technology | #RM3002 |
| Anti-mouse CD8a | eBioscience | 17-0081-82 |
| Anti-mouse CD4 | eBioscience | 48-0042-82 |
| Anti-mouse IFN-gamma | eBioscience | 12-7311-82 |
| Anti-mouse Foxp3 | eBioscience | 72-5775-40 |
| Anti-mouse CD11b | eBioscience | 48-0112-82 |
| Anti-mouse Ly6C | eBioscience | 17-5932-82 |
| Anti-mouse Ly6G | eBioscience | 11-9668-82 |
| Recombinant IFN-I (IFN-β) | R&D systems | 8234-MB |
| anti-mouse IFNAR | BioXcell | BP0241 |
| isotype IgG | BioXcell | BE0083 |
